# Unconventional Parahydrogen-Induced Hyperpolarization Effects in Chemistry and Catalysis: From Photoreactions to Enzymes

**DOI:** 10.1021/acscatal.4c07870

**Published:** 2025-04-04

**Authors:** Andrey N. Pravdivtsev, Ben J. Tickner, Stefan Glöggler, Jan-Bernd Hövener, Gerd Buntkowsky, Simon B. Duckett, Clifford R. Bowers, Vladimir V. Zhivonitko

**Affiliations:** † Department Section Biomedical Imaging, Molecular Imaging North Competence Center (MOIN CC), Department of Radiology and Neuroradiology University Medical Center Kiel, Kiel University, Am Botanischen Garten 14, 24118 Kiel, Germany; ‡ Centre for Hyperpolarization in Magnetic Resonance (CHyM), Department of Chemistry University of York, Heslington, YO10 5NY, United Kingdom; § Max-Planck-Institute for Multidisciplinary Sciences, Am Faßberg 11, 37077 Göttingen, Germany; ∥ Center for Biostructural Imaging of Neurodegeneration (BIN), Von-Siebold-Str. 3a, 37075 Göttingen, Germany; ⊥ Advanced Imaging Research Center, The University of Texas Southwestern Medical Center, Dallas, Texas 75390, United States; # Eduard-Zintl-Institut für Anorganische und Physikalische Chemie, Technische Universität Darmstadt, Peter-Grünberg-Str. 8, D-64287 Darmstadt, Germany; ∇ Department of Chemistry and National High Magnetic Field Laboratory, University of Florida, Gainesville, Florida 32611, United States; ○ NMR Research Unit, University of Oulu, P.O. Box 3000, Oulu 90014, Finland

**Keywords:** parahydrogen, catalysis, hyperpolarization, mechanisms, NMR

## Abstract

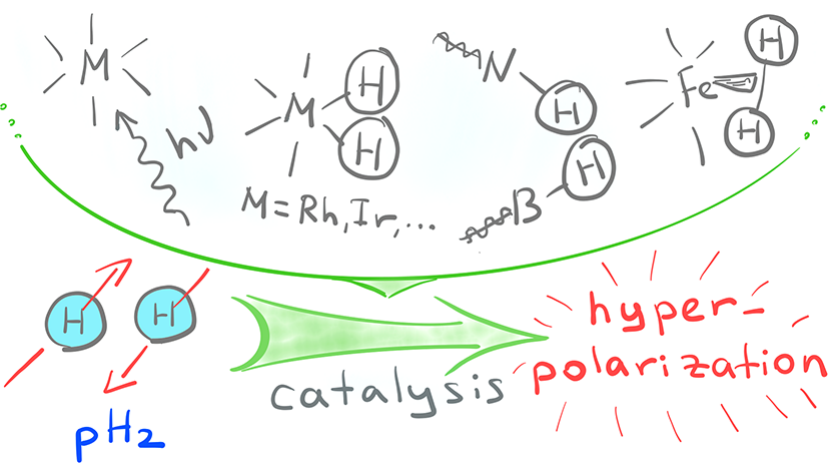

Nuclear spin hyperpolarization utilizing parahydrogen has the potential for broad applications in chemistry, catalysis, biochemistry, and medicine. This review examines recent chemical and biochemical insights gained using parahydrogen-induced polarization (PHIP). We begin with photoinduced PHIP, which allows the investigation of short-lived and photoactivated catalysis. Next, we review the partially negative line effect, in which distinctive line shape helps to reveal information about rapid exchange with parahydrogen and the role of short-lived catalytic species. The NMR signal enhancement of a single proton in oneH-PHIP is discussed, challenging the underpinning concept of the necessity of pairwise hydrogenation. Furthermore, we examine metal-free PHIP facilitated by frustrated Lewis pair molecular tweezers and radicaloids, demonstrating alternative routes to hydrogenation. Although symmetric molecules incorporating parahydrogen are NMR silent, we showcase methods that reveal hyperpolarized states through post-hydrogenation reactions. We discuss chemical exchange processes that mediate polarization transfer between parahydrogen and a molecular target, expanding the reach of PHIP without synthesizing specialized precursors. We conclude this review by highlighting the role of PHIP in uncovering the H_2_ activation mechanisms of hydrogenases. By providing a detailed review of these diverse phenomena, we aim to familiarize the reader with the versatility of PHIP and its potential applications for mechanistic studies and chemical analysis.

## 1 Introduction

Without hesitation, nuclear magnetic resonance (NMR) is one of the leading and most widely used spectroscopic methods in chemistry. This success is due to its broad analytical capabilities and noninvasive nature, enabled by the minuscule magnetic moments associated with the nuclear spins. Despite its ubiquitous role in molecular characterization and analysis, NMR suffers from low sensitivity, as only a tiny fraction of all available nuclear spins effectively contributes to the total NMR signal. Consequently, combinations of time-consuming signal averaging, concentrated samples (>mM), and elaborate equipment/hardware are often necessary. Addressing these challenges is the focus of much attention, and the scope of NMR applications is constantly expanding through the introduction of more sensitive NMR instruments with higher magnetic fields,^1^ cryoprobes,^2^ ultrafast sequences,^3^ and nuclear spin hyperpolarization methods.^4^

In this review, we focus on one of many hyperpolarization methods that increase nuclear spin polarization using various physical and chemical effects. Such processes create transient non-Boltzmann population distributions across closely spaced nuclear spin energy levels. This effect is highly beneficial as NMR signal enhancements of several orders of magnitude can be obtained. For example, at a clinical field strength of 1.5 T, only 0.000125%, about one in 800,000 ^13^C nuclei, is effectively detected (thermally polarized under ambient conditions). When the system is fully polarized, all ^13^C spins contribute to the signal, enhancing the signal—and thus the sensitivity—by more than 5 orders of magnitude. Hence, hyperpolarization is a versatile tool that aids the magnetic resonance detection of molecules at low concentrations or short lifetime, with diverse applications in biomedical imaging,^5−8^ protein studies,^9−11^ composition analysis^12,13^ and catalysis.^14−16^

Parahydrogen-induced polarization (PHIP), introduced in the 1980s,^17−19^ exploits the alignment of the nuclear spins in the singlet state spin isomer of molecular hydrogen, parahydrogen (pH_2_).^20^ pH_2_ reflects the lowest energy spin isomer of dihydrogen and can be easily prepared in an almost pure state by cooling hydrogen gas to 20 K or below,^21−24^ though 77 K is often used for practical simplicity and yields a pH_2_ enrichment of about 50%.^25−27^ Para-enriched H_2_ gas can typically be stored for weeks at ambient temperatures and used on demand to generate nuclear spin hyperpolarization. However, as the para-state is associated with singlet nuclear spin order, it does not yield any NMR signal and is considered “NMR silent”; further chemical reactivity breaking the symmetry of pH_2_ is required to enhance the NMR signal.^17−19^

**Figure 1:**
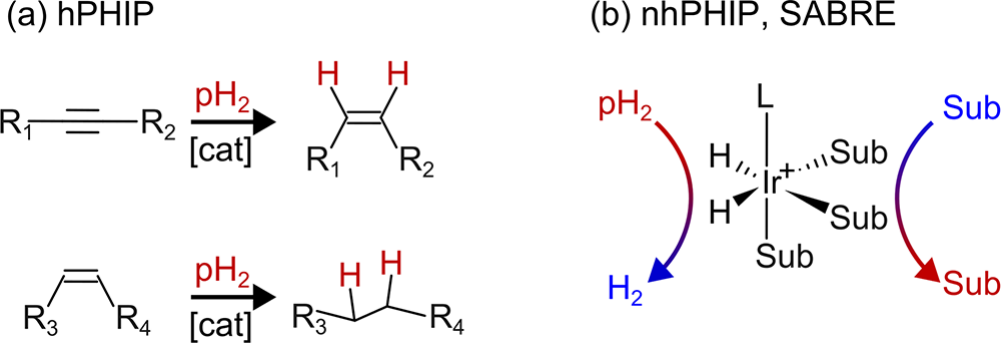
hPHIP and SABRE. Schematics of (a) hPHIP, whereby parahydrogen (pH_2_) is added to an unsaturated bond in the presence of a catalyst, [cat], and (b) SABRE, where a ligating substrate, Sub, and pH_2_ undergo reversible exchange at a catalyst. The typical SABRE auxiliary ligand L is IMes = 1,3-bis(2,4,6-trimethylphenyl)-1,3-dihydro-2*H*-imidazol-2-ylidene.

The detailed spin dynamics-based theory underlying PHIP and related phenomena are well described elsewhere.^20,28−265^ Here, we aim to provide an accessible narrative that, while linking appropriate physicochemical descriptions, does not overburden the reader with overly advanced spin physics. In classical PHIP experiments,^17−19^ which are also termed hydrogenative PHIP (hPHIP), two protons of pH_2_ are typically added at vicinal positions to double or triple CC bonds in an unsaturated substrate through catalytic hydrogenation (Figure 1a). As the spin states in the newly formed molecule evolve, the ^1^H NMR signal can be significantly enhanced. In this regard, two experimental schemes are distinguished: parahydrogen and synthesis allow dramatically enhanced nuclear alignment (PASADENA), where the hydrogenation process takes place at a high magnetic field,^18^ and adiabatic longitudinal transport after dissociation engenders net alignment (ALTADENA), where hydrogenation occurs at low-field.^31^ In both cases, the resultant ^1^H hyperpolarization can be used as is or transferred to another nucleus, such as ^13^C and ^15^N, e.g., for observation to benefit from longer relaxation times, no background signals, and greater chemical shift dispersion relative to ^1^H. While hPHIP has been most commonly achieved by homogeneous Rh or Ru catalysts,^32−35^ other metals^36−41^ and heterogeneous catalysts have also exhibited PHIP,^42,43^ although typically with smaller polarization than homogeneous systems.

For alkyne substrates, *cis*-vicinal hydrogenation is the most common type of hydrogenation achieved by homogeneous metal catalysts, and, therefore, it is a common reaction used in hPHIP (Figure 1a). Recently, however, *trans*-vicinal hydrogenation was turned into a dominant pathway to unlock direct hyperpolarization of fumarate^44,45^—an intermediate in the citric acid cycle—and a marker of cell necrosis.^46^ In contrast, heterogeneous catalysts (e.g., supported metal nanoparticles) typically yield low hydrogenation selectivity as the reaction mechanism can involve multiple metal sites that deliver their hydrogen atoms to various positions in the product, either directly or reversibly.^15,43,49,50^ The convenience of using heterogeneous catalysts is now facilitating the production of hyperpolarized gases as pH_2_-enhanced contrast agents for lung MRI.^47,48^

In general, hPHIP experiments can also be used to quantify the precise stereo and regioselectivity in such processes through the signal amplification effect.^49−52^ Regardless of the hydrogenation mechanism, a crucial requirement to observe hPHIP is the pairwise addition of H_2_ to a metal catalyst or unsaturated precursor. In other words, both ^1^H spins of the same pH_2_ molecule must end up in the same product molecule to retain their spin correlation. This requirement has provided significant insight into metal-based oxidative addition reactions.^53^

**Figure 2:**
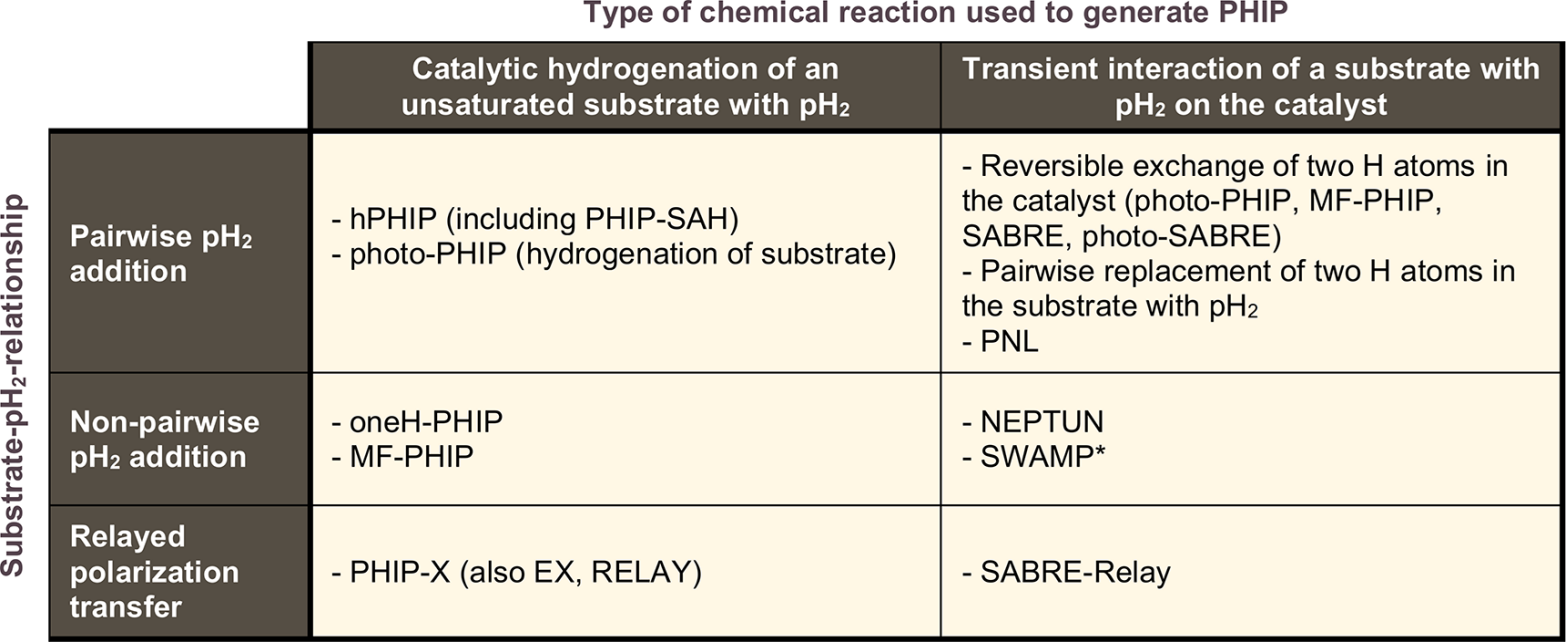
Classification of chemical effects that lead to PHIP. The primary scope of this review is to describe the pH_2_-derived hyperpolarization effects except those of routine hPHIP and SABRE, which are well-reviewed. *Preliminary assignment based on current data. *Adapted with permission from Emondts et al*.^109^*Copyright 2018 John Wiley and Sons*.

An alternative to hPHIP is non-hydrogenative PHIP (nhPHIP, Figure 2), with its most popular form called signal amplification by reversible exchange (SABRE, Figure 1b).^54^ In SABRE, pH_2_ and a substrate ligate transiently with a metal center. Depending on the experimental conditions, *J*-couplings or cross-relaxation can then drive the conversion of the spin alignment of the pH_2_-derived spins into the spin polarization of the transiently bound substrate in the resulting complex. Subsequent dissociation of this ligand results in a chemically unaltered but hyperpolarized free substrate in solution,^55^ allowing for multiple contacts and continuous polarization of the same molecule.^56−58^ Typically, the most efficient polarization transfer in SABRE is reached when there is a match between certain energy levels in the SABRE complex spin system, and this can be readily achieved by selecting appropriate DC or AC magnetic fields for the experiment.^59−62^ Such a matching condition is more precisely termed level anticrossing (LAC), and its theoretical description in the context of hyperpolarization is well-reviewed.^63^

Several specific requirements must be met for molecules to become hyperpolarized by PHIP. For hPHIP, these include the presence of an unsaturated functionality (typically alkene or alkyne groups) that can accept pH_2_. The strategy to hyperpolarize molecules that do not possess an appropriate unsaturated bond^64−71^ or ligating functionality^72,73^ is to add such a function on a side arm. The resulting side arm is then released after hydrogenation and hyperpolarization transfer by hydrolysis (e.g., PHIP-SAH).^65−71^ For SABRE, the substrate and pH_2_ must ligate to a metal center, form spin–spin interactions, and dissociate. Therefore, such substrates often contain an electron-donating nitrogen site.^54,74^ However, the ongoing design of new catalysts has significantly expanded the types of molecules amenable to SABRE to include biologically significant O-donor ketoacids.^75^ Other studies have seen sulfur, phosphorus, and silicon donor sites employed.^76−78^

Collectively, hPHIP,^47,79−84^ PHIP-SAH,^85−87^ and SABRE^88,89^ have led to *in vivo* metabolic imaging applications. The translation from optimization of PHIP to *in vitro* and *in vivo* studies is not straightforward but has accelerated in recent years, demonstrating PHIP as a viable route to produce preclinical and clinical hyperpolarized agents in liquid and gas phases competitive with other approaches such as dissolution dynamic nuclear polarization (dDNP)^85^ or spin-exchange optical pumping (SEOP).^90−92^ Advances in PHIP for *in vivo* imaging have been well-reviewed.^4,93−96^ This progress is underpinned by several advancements in the instrumentation associated with PHIP delivery.^97^

In addition to the relatively well-known protocols of hPHIP and SABRE, there are other exciting effects that, although less well appreciated, more generally illustrate the unique properties of pH_2_, which in turn provide valuable insight into both chemical and catalytic reactivity.^29^ For example, one less common type of hPHIP is geminal hydrogenation, where two protons of pH_2_ bind to the same carbon.^98,99^ There are other variations of the PHIP effect, such as when the catalyst itself interacts with pH_2_ and becomes transiently polarized.^100−104^ Other cases include pairwise replacement of two substrate protons with a proton pair from pH_2_ with no net hydrogenation,^16,49,105,106^ the addition of only one proton from a pH_2_ molecule to the substrate (oneH-PHIP)^107^ (after the formation of an intermediate by pairwise addition) or even hydrogenation accompanied by oligomerization.^108^

This review will focus on such more unusual PHIP effects beyond PASADENA, ALTADENA, and SABRE (Figure 2). We will discuss these phenomena and their mechanisms, seeking to promote new analytical applications and provide valuable insight into the underlying chemical interactions. In this regard, we will discuss photoinduced PHIP and SABRE, partially negative line (PNL) effects and their implications for the analysis of short-lived intermediates, oneH-PHIP effects in hydroformylation, the various mechanisms of hyperpolarization of water using pH_2_, PHIP effects in metal-free hydrogenation (MF-PHIP) reactions, secondary transformation which reveal hidden PHIP, chemically relayed polarization transfer, and PHIP in enzymatically catalyzed hydrogenation reactions.

## 2 Unconventional Variants of PHIP

### 2.1 Photo-PHIP and Photo-SABRE

**Figure 3:**
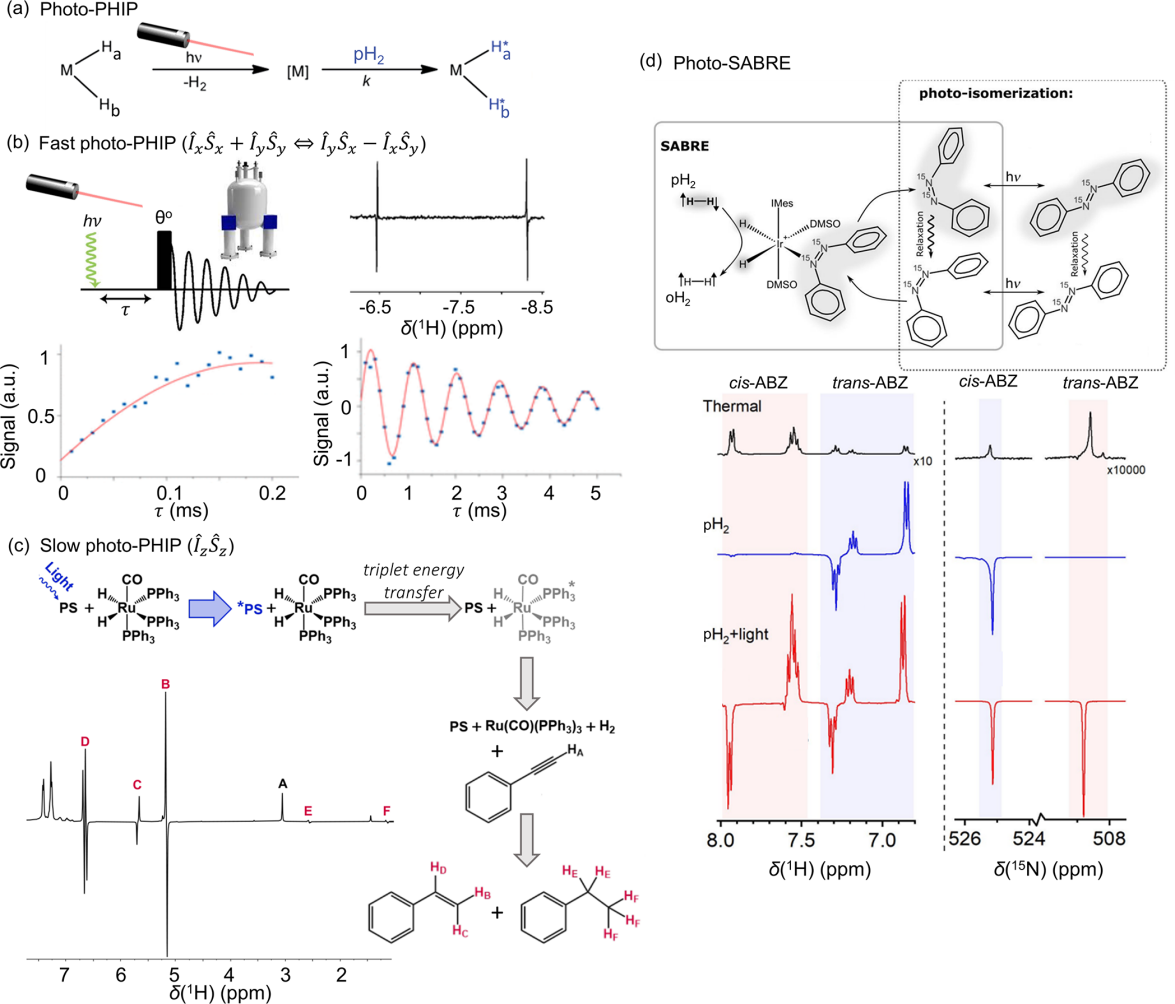
Examples of photo-PHIP. (a) The outline of a photo-PHIP experiment in which light-induced ligand dissociation creates a metal complex [M] that can add pH_2_ to create an inequivalent metal dihydride species with PHIP-enhanced hydride NMR signals. (b) Depiction of laser-pump NMR-probe spectroscopy in which laser irradiation stimulated H_2_ – pH_2_ exchange followed by NMR detection (where θ is typically 45° or 90°), with a variable delay between the two (top left). The NMR spectrum at the top right is a single-laser shot, single 90° RF pulse, pH_2_-enhanced ^1^H{^31^P} NMR spectrum of the hydride region of [Ru(PPh_3_)_3_(CO)(H)_2_] with τ = 0.05 ms (broadband ^31^P decoupling). When the delay is on the millisecond time scale, the zero quantum coherences of pH_2_ in the chemically inequivalent dihydride complex can evolve due to the chemical shift difference from unobservable *Î*_*x*_*Ŝ*_*x*_ + *Î*_*y*_*Ŝ*_*y*_ to observable *Î*_*y*_*Ŝ*_*x*_ – *Î*_*x*_*Ŝ*_*y*_. The lower kinetic traces show the hydride signal integral at δ ∼ −6.5 ppm from ^1^H{^31^P} NMR spectra of [Ru(PPh_3_)_3_(CO)(H)_2_] (1 × 10^–3^ M, 3 bar pH_2_) as a function of τ. Experimental points are shown in blue with fitted red lines. This spin evolution can give information about kinetic hydrogen addition rates. (c) Slow photo-PHIP leading to hyperpolarized styrene and ethylbenzene after [Ru(H)_2_(PPh_3_)_3_(CO)] and an iridium photosensitizer (PS) are irradiated at 420 nm in DCM-*d*_2_ at 298 K.^127^ In these cases, delays between laser irradiation and NMR pulse acquisition are on the order of seconds. Consequently, ZQC coherences are not observed, which is similar to the traditional PASADENA effect. (d) Depiction of photo-SABRE in which a SABRE-catalyst hyperpolarizes *cis*-azobenzene (ABZ) followed by light irradiation to switch between *cis* and *trans* isomers of ABZ (upper). Hyperpolarized molecules are shown with a gray background.^128^ Example ^1^H (lower left) and ^15^N (lower right) NMR spectra were recorded after 10 min of light irradiation of a sample containing 56 mM of ^15^N_2_-ABZ in CD_3_OD with 1 mM of [IrCl(COD)(IMes)] and 200 mM DMSO-*d*_6_ at 9.4 T. SABRE polarization transfer to ^1^H was performed at 200 nT and that to ^15^N, at 400 nT. Signals for *cis*-ABZ are shown with a red background, with those of *trans*-ABZ with a blue background. *(a) Adapted with permission from Procacci et al*.^114^*Copyright 2016 Royal Society of Chemistry. This publication is licensed under CC BY-NC 3.0. (b) Adapted from Torres et al*.^113^*Copyright 2014 American Chemical Society. This publication is licensed under CC-BY. (c) Adapted from Brown et al*.^127^*Copyright 2022 American Chemical Society. This publication is licensed under CC-BY 4.0. (d) Adapted with permission from Kiryutin et al*.^128^*Copyright 2024 John Wiley and Sons*.

Breaking the bond in pH_2_ is a critical step in any PHIP-type experiment, as it unlocks the spin order of pH_2_, ideally without losing the initial spin alignment. This step is most commonly associated with the oxidative addition of pH_2_ to a metal center. An essential feature of this step, therefore, is the involvement of metal complex species with appropriate electron configurations (preferably diamagnetic) that both prevent rapid nuclear spin relaxation and support the formation of two new metal–hydride bonds. Accordingly, many stable complexes must undergo ligand loss before such a process occurs. This has given rise to studies where light irradiation stimulates ligand loss from a stable metal complex to generate a reactive intermediate that can react rapidly with pH_2_ (Figure 3a).^110−116^ Consequently, hyperpolarized metal complexes are formed in a process that is somewhat analogous to hPHIP, which involves pH_2_ addition to a stable 16-electron transition metal precursor. However, combining photochemistry with PHIP, termed photo-PHIP, can create magnetic states that differ from those created under conventional PASADENA-type PHIP.^18,117,118^ This photochemical approach has been used to study rapid kinetics^111,114^ and detect reaction intermediates or products.^110,114,119^ The examples of photo-PHIP typically involve Ir and Ru carbonyl precursors containing phosphine, diphosphine, or diarsine ligands.^115,116,119,120^

To introduce photo-PHIP in further detail, it is convenient to provide theoretical considerations that illustrate the basic differences between traditional and photochemistry-assisted approaches. For reference, pH_2_ exists as a nuclear spin singlet state that is defined by the density matrix described in eq 1
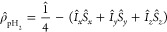
1where *Î*_*i*_ and *Ŝ*_*i*_ (*i* = *x*, *y*, *z*) are spin operators which refer to identical atoms in pH_2_. This state consists of the longitudinal two-spin order *Î*_*z*_*Ŝ_z_*, and the in-phase zero-quantum coherence (ZQC) *Î*_*x*_*Ŝ_x_* + *Î*_*y*_*Ŝ_y_*. In hPHIP, numerous catalytic hydrogenation steps, or reversible H_2_ exchange in SABRE, commonly create weakly coupled metal-dihydrides. In this case, the in-phase ZQC oscillates due to the periodic transformation into the out-of-phase ZQC state, *Î*_*y*_*Ŝ_x_* – *Î*_*x*_*Ŝ_y_*. As a result, it averages to zero over the time of thermally initiated reactions,^121^ unless strong proton decoupling is applied^82,122^ or experiments are performed at low magnetic fields when metal-dihydrides are strongly coupled.^123^

In contrast, the photo-PHIP approach involves short laser-induced photodissociation (on the order of nanoseconds) to create a vacant ligand site such that rapid reaction with pH_2_ is possible (Figure 3a). As a consequence of the relatively facile pH_2_ addition (faster than 1/*v*_ZQC_ such that ZQC does not have enough time to oscillate) all terms in eq 1 are preserved through the hydrogenation reaction even though the singlet state is not stationary in the dihydride product complex.^116^ Due to this, the ZQC oscillation is observable in the subsequent ^1^H NMR measurements (Figure 3b). For two weakly coupled spins, this oscillation of ZQC occurs at a frequency equal to the difference between the Larmor frequencies of the two nuclei, *v*_ZQC_ = |*v*_I_ – *v*_S_|, where *v*_I_ and *v*_S_ are the Larmor frequencies of the respective spins I and S. Typically, in photo-PHIP systems, there are strong interactions between hydride protons and ^31^P of spectator phosphine ligands, which additionally contribute to *v*_ZQC_.

At the instant the symmetry of the initial pH_2_ is broken in forming the newly created hydride ligands (the time after the laser pulse is essentially τ = 0 for a rapid reaction), the newly formed spin system cannot be perturbed by a hard excitation RF pulse as it is instantly formed from the NMR silent singlet state (Figure 3b). Consequently, this situation does not lead to an observable NMR signal. However, as the transverse ZQC term (eq 1) evolves under chemical shift and coupling, an NMR-observable spin state arrangement is created that can be probed directly with hard pulses (typically 45° or 90°, Figure 3b).^112,122,124^ This approach was used to study the evolution of dihydride singlet order for a wide range of Ru and Ir complexes with a variable τ between laser irradiation and NMR detection.^112^ This type of laser-pump NMR-probe experiment, conducted over a microsecond-to-millisecond delay time scale, allowed the observation of reactivity in an approach analogous to other laser-based UV and IR time-resolved spectroscopies, which was only possible by marrying laser-induced ligand dissociation with a pH_2_ addition step (Figure 3b).^110−116^ Multiple laser pulses or continuous wave laser irradiation over times much longer than 1/*v*_ZQC_ have also been employed.^114,116^ However, in these cases of slow hyperpolarization preparation, spin order is averaged across the light irradiation time, which leads to an effect analogous to the time-averaged PASADENA effect as the ZQC terms are lost (Figure 3c).

In contrast to hard pulse excitations, the selective RF excitation of one of the chemically inequivalent hydride ligands in the complex can lead to the observation of strong NMR signals without the need for further spin-state evolution after the laser pulse.^125^ Alternatively, adiabatic RF pulses can convert the singlet spin order into observable magnetization of one or two protons.^124,126^

The analysis process required to extract information about the kinetics of pH_2_ addition in photo-PHIP experiments is complex.^111,113,124^ For example, in cases where the pH_2_ addition rate is on the same order of magnitude as the frequency of ZQC evolution, *v*_ZQC_, only partial averaging occurs. This can lead to apparent phase shifts in the signal oscillation that contain quantitative information about the pH_2_ addition rate.^114^ However, if product formation is much faster than the rate of ZQC evolution, then only an upper bound for the H_2_ addition rate can be determined. On the other hand, if product formation is much slower than the characteristic frequencies of the spin system evolution, the ZQC oscillations are no longer observed due to complete averaging. The latter case is similar to traditional PHIP in which thermally controlled reactions (rather than laser-induced) build up the number of H_2_ addition products over a longer time window (ca. seconds).^111^

The photo-PHIP method has been applied to measure the H_2_ addition rate of [Ir(I)(PPh_3_)_2_(CO)], which is formed from laser-induced H_2_ dissociation from [Ir(I)(H)_2_(PPh_3_)_2_(CO)] (Figure 3b).^114^ Notably, the obtained rate constants were comparable to those measured using flash photolysis coupled with optical spectroscopy.^114^ However, unlike flash photolysis, NMR has additional chemical resolution that enables a more detailed analysis of chemical reactions, which is especially important for mixtures of photoactive substrates.

Approaches of this type have also been used to study ruthenium arsine complexes that act as alkyne hydrogenation catalysts as catalytic activity is observed after photolysis, and many intermediates and species involved in the catalytic cycle are detected and characterized with the help of photo-PHIP-enhanced signals.^115^ Due to the lower reaction rate of these catalysts, these types of examples can be called slow photo-PHIP. The delay between laser irradiation and NMR signal acquisition is much longer (on the order of seconds). ZQC completely decays, revealing enhanced antiphase NMR signals analogous to those in traditional PASADENA experiments without amplitude oscillations. Initiating hydrogenation catalysis with a laser pulse allows access to hyperpolarized species inaccessible in thermal reactions (without laser-induced dissociation), as thermal conditions are insufficient to enable ligand dissociation, which must occur before pH_2_ addition for many metal complexes. One recent example also involved the use of an irradiated iridium photosensitizer to produce excited [Ru(H)_2_(PPh_3_)_3_(CO)], which in turn stimulated H_2_ dissociation to form [Ru(PPh_3_)_3_(CO)]. Subsequent hydrogenation of phenylacetylene with pH_2_ yielded PHIP-enhanced styrene (single hydrogenation) and ethylbenzene (double hydrogenation) with ^1^H NMR signals for these organic photoactivated hydrogenation products enhanced by up to 1630 times at 9.4 T (Figure 3c).^127^

While photoactivated PHIP has several examples in general, few examples of photoactivated SABRE exist. SABRE typically relies on iridium catalysts that reversibly exchange H_2_ under thermal conditions. Rational catalyst design is, however, often used to improve the efficiency of SABRE by tuning ligand exchange rates or controlling the binding of particular target substrate molecules.^16^ One example of a type of photo-SABRE involved azobenzene, with light irradiation of the target ligand rather than the metal catalyst. This approach used light irradiation to switch azobenzene between *cis* and *trans* conformations, controlling SABRE activity as only *cis*-azobenzene has appropriate geometry for ligation to the iridium SABRE catalyst (Figure 3d).^128^ Upon hyperpolarization of its ^15^N nuclei using a traditional SABRE approach, light-induced isomerization locked the polarization within *trans*-azobenzene as it does not interact with the metal center, allowing for prolonged polarization lifetimes. Another benefit was that the *trans* isomer also formed a long-lived spin state with a lifetime of *ca*. 25 min, which means it can act as a store of polarization. While this example utilized a photoswitchable target, in the future, light-controlled catalysts could be exploited for SABRE, where photoactivation initiates either H_2_ or substrate exchange within the metal coordination sphere. Indeed, while this review was provisionally accepted, an example of the latter was reported.^266^ Developing such photo-SABRE approaches would allow the precise tuning of the scalar coupling network within the transient active catalyst to match the ligand exchange rate, thereby optimizing polarization transfer to the bound substrate. By employing photochemical control, such systems could achieve polarization levels closer to the theoretical maximum than those currently achieved through thermally driven exchange. Moreover, rather than requiring a diverse range of catalysts to accommodate different substrates and conditions, a single, photoswitchable catalyst could potentially be used. This would enhance both efficiency and selectivity while simplifying synthetic needs. Given these potential advantages, developing new classes of photo-SABRE catalysts reflects a promising avenue for advancing this hyperpolarization technique.

### 2.2 Reversible H_2_ Exchange and the Partial Negative Line (PNL) Effect

**Figure 4:**
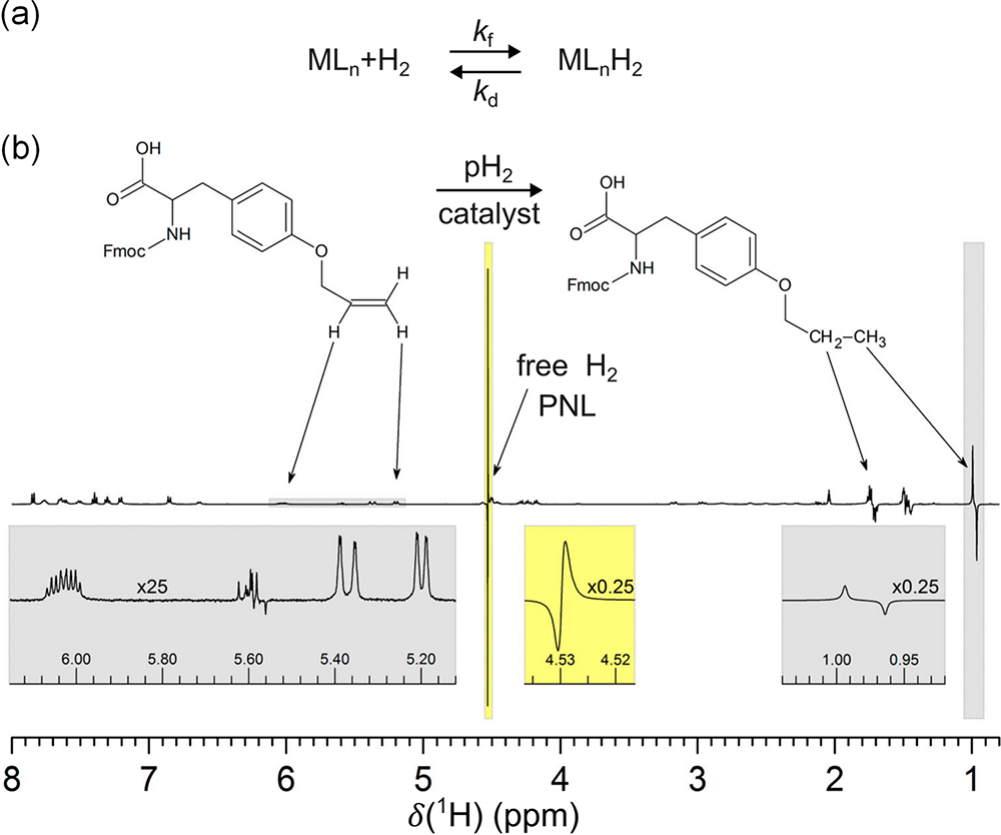
Partial Negative Line effect induced by a catalyst–pH_2_ interaction. (a) Chemical exchange of pH_2_ with the metal center ML_n_ of the catalyst results in the PNL effect on free H_2_. (b) ^1^H PHIP NMR spectrum recorded during the hydrogenation of Fmoc-O-allyl-tyrosine (left) to Fmoc-O-propyl-tyrosine (right) with pH_2_, showing a strong PNL signal for free H_2_ at 4.53 ppm. *Adapted with permission from Kiryutin et al*.^130^*Copyright 2017 American Chemical Society*.

Intra- and intermolecular chemical exchange can have a strong influence on the appearance of the resonances in an NMR spectrum, inducing line broadening and line shape distortions. Consequently, NMR line shape analysis can be employed as an analytical tool to monitor the dynamics of transient catalytic species. In the context of PHIP, such behavior is generally relevant to molecular dihydrogen complexes interacting reversibly with pH_2_ and hence dissolved dihydrogen (Figure 4a), as well as other weakly interacting ligands. At the same time, since these processes involve pH_2_, unlocking its magnetism introduces novel phenomena that cannot be observed with normal H_2_. One intriguing effect of this kind was independently observed as an enhanced antiphase signal for dissolved H_2_ by Zhivonitko et al.^129^ during the investigation of pH_2_ activation with *ansa*-aminoboranes and by Kiryutin and Sauer et al.^130^ during the investigation of the PHIP enhancement of small oligopeptides (Figure 4b). This phenomenon is now referred to as the partially negative line (PNL) shape of the dihydrogen NMR signal.

The observation of the PNL effect is unusual because dihydrogen is a symmetric molecule that should exhibit only a single resonance in ^1^H NMR (A_2_ spin system). However, the antiphase character of the H_2_ signal in PHIP experiments implies the presence of two resonances (Figure 4b).^130^ Kiryutin et al. showed that the PNL effect is independent of the presence of a substrate and occurs via the transient interaction of the pH_2_ molecules with the catalyst under fast chemical exchange.^130^ Using the double quantum coherence filter variant of the only parahydrogen spectroscopy (OPSY) NMR pulse sequence,^131−134^ it was demonstrated that the PNL effect results from the longitudinal two-spin order, *Î*_*z*_*Ŝ*_*z*_, originating from pH_2_ (eq 1). Normally, this term is NMR-invisible for a pair of magnetically equivalent protons in H_2_. However, the rapid exchange between free H_2_ and two chemically inequivalent hydride ligands in a transient catalyst–dihydrogen complex (Figure 4a) leads to a slight frequency difference for two ^1^H resonance components of free H_2_. In addition, the interaction of pH_2_ with the catalyst promotes so-called singlet–triplet mixing^118,135−137^ when pH_2_-originating protons become transiently inequivalent in the complex. This mixing, accompanied by the hydride–H_2_ exchange, leads to the averaging of the ZQC terms derived from the initial pH_2_ singlet spin order (eq 1 and discussion thereof) to zero, while the longitudinal term, *Î*_*z*_*Ŝ*_*z*_, is preserved in both free H_2_ and bound hydride ligands. Altogether, PASADENA-type exchange broadened antiphase signals are observed for dihydride ligands, while the H_2_ signal is revealed as a superposition of two enhanced PNL resonance lines with opposite phases and slightly shifted frequencies. In the original work, the former species were difficult to detect directly due to heavy line broadening and their low concentration, while PNL was clearly visible and therefore useful.^130^

**Figure 5:**
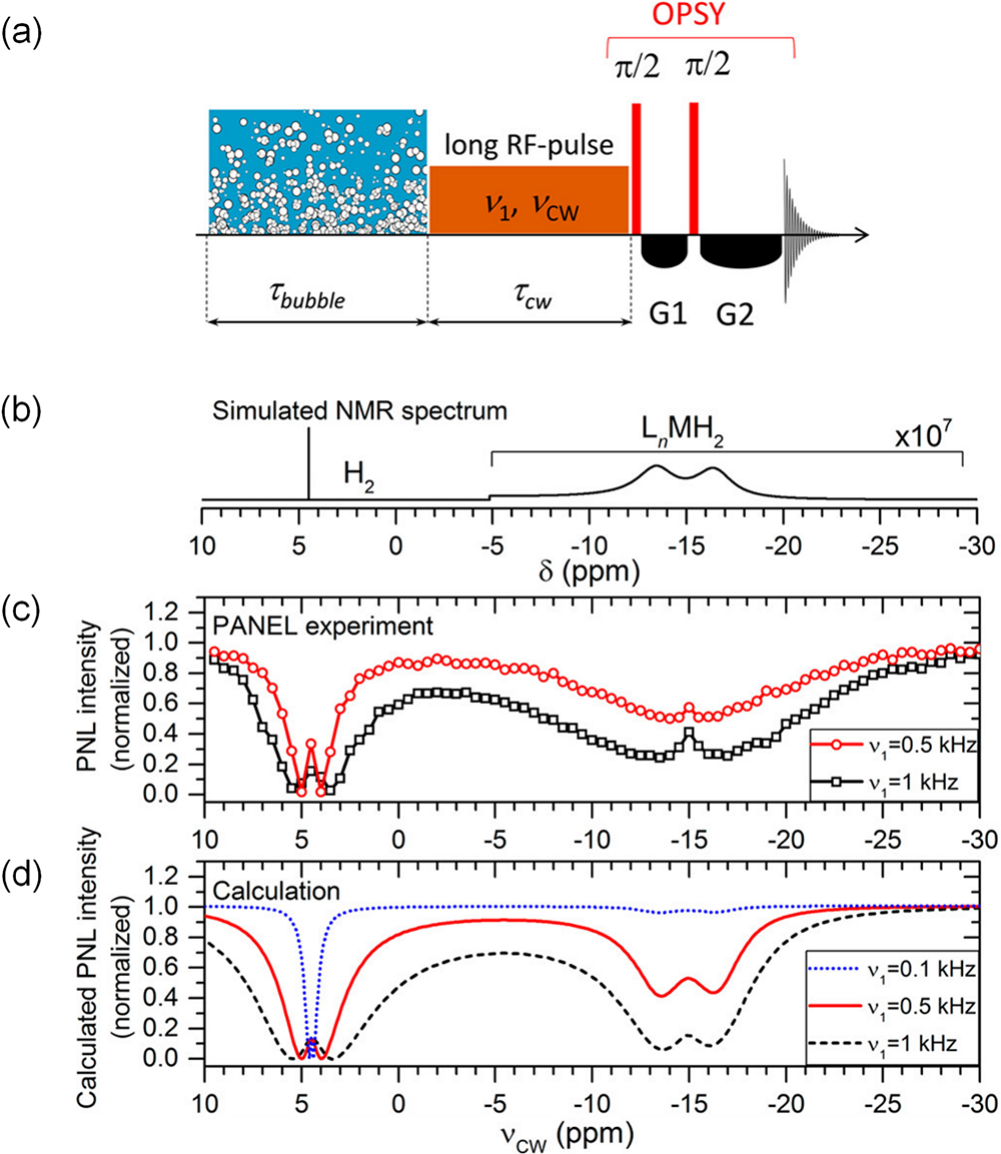
PANEL – A combination of the CEST method and the PNL effect boosts the sensitivity of catalytic intermediate detection. (a) Scheme of the PANEL (partially negative line) experiment for indirectly detecting the hidden hydrogen catalyst complex (L_n_MH_2_). (b) Calculated NMR spectrum of the hidden complex, magnified by a factor of 10^7^. (c) Experimental and (d) simulated PANEL spectra showing the signal of the hidden complex (lines at −16.5 ppm and −13.5 ppm). *Adapted with permission from Kiryutin et al*.^130^*Copyright 2017 American Chemical Society*.

Based on this explanation of the PNL effect, the partially negative line experiment (PANEL)^130^ was developed (Figure 5a), which employed continuous-wave low-power radio frequency irradiation to detect transient species indirectly by observing the change in the PNL signal of free H_2_. When the frequency of the continuous radiofrequency pulse is in resonance with one of the hydrogens bound to the short-lived catalyst or free hydrogen, this nucleus is saturated, and the PNL is strongly affected. This experiment is similar to chemical exchange saturation transfer (CEST) NMR experiments, which have also been used to study short-lived exchanging intermediates.^138^ Using PANEL, a sensitivity gain of at least 3 orders of magnitude, compared to routine NMR experiments, is achievable (Figure 5b). As with CEST, the sensitivity and spectral resolution of PANEL depend on the amplitude of the RF saturation field.^139^ Since hydride nuclei often resonate at chemical shifts below −10 ppm, while the PNL signal for H_2_ appears at around 4.5 ppm, the associated resonance separations reach several kHz in high magnetic fields (e.g., 5.8 kHz at 9.4 T). Consequently, RF pulses of 1 kHz amplitude can be applied without intrinsically distorting the H_2_ signal. Hence, when used to probe the hydride region, the PANEL experiment can detect otherwise invisible intermediates with lifetimes of less than 1 ms. The achievable spectral resolution of the PANEL depends on the RF power used. Resolutions of 1 ppm are achievable by employing RF amplitudes of ca. 100 Hz.

The presence of two signals of the same intensity at −16.5 ppm and −13.5 ppm in the PANEL spectrum (Figure 5c) points to the sizable chemical shift difference between two inequivalent hydrides and the corresponding complex asymmetry. In such a weak coupling case (|ω_I_ – ω_S_|/2π ≫ |*J*_IS_|) the H_2_ resonance splitting that leads to PNL is given by eq 2(130)
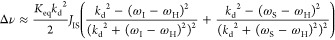
2where ω_I_, ω_S_, ω_H_ are frequencies of the spins in the complex (I and S) and of free dihydrogen (H), *J*_IS_ is the constant of spin–spin interaction in the complex, *K*_eq_ = *k*_f_[ML_n_]/*k*_d_ is the equilibrium constant of the binding of dihydrogen to the complex, *k*_d_ is the dissociation rate constant, and *k*_f_ is the formation rate constant.

The same theoretical model of PNL^130^ reproduced the experimental NMR line-shapes, the nutation angle dependence, and the dependence on the frequency of the resonance position of the PNL. It also permitted the determination of chemical shift values for exchanging protons in the transient complex and the sign of the scalar coupling constant between those protons. Typically, hydride resonances of transient complexes are hardly observable directly in ^1^H NMR, whereas PNL allows for indirect but sensitive detection of their presence.^130^ In parallel to that, Johnson et al.^140^ observed PNL and then managed to detect a hyperpolarized dihydrogen complex by cooling the catalyst sample with pH_2_ down to 238 K, confirming the presence and rapid exchange of the dihydrogen. The observation of reaction intermediates is highly valuable as such species are difficult to discern in standard ^1^H NMR experiments due to their low signal intensity and broad line widths. These techniques were later applied to study several exchange pathways with pH_2_.^141^

Bernatowicz et al.^142^ proposed an alternative explanation for the creation of PNL, which suggests it is caused by residual dipolar couplings (RDCs) stemming from the partial ordering of the hydrogen molecules in the external magnetic field. However, further experimental studies into this mechanism are warranted.

Czarnota et al.^143^ and Alam et al.^144^ investigated the conditions for a PNL effect employing iridium complexes or metal–organic frameworks (MOFs). PNL effects were found in the catalytic hydrogenation of eptifibatide, a disintegrin derivative based on a protein from the rattlesnake venom,^145^ or trivinyl orthoacetate,^99^ as well as in SABRE studies employing Zintl cluster-supported rhodium centers^146^ or nickel diazadiphosphacyclooctane complexes.^141^ Finally, PNL effects are also a possible loss channel for hyperpolarization in hPHIP or SABRE applications.^118,136,137,147^ PNL not only allows for ultrasensitive detection of intermediates but can also serve in the future as a tool to monitor spin dynamics and, thus, chemical kinetics in intermediate complexes.

### 2.3 OneH-PHIP

Pairwise addition of pH_2_ to an unsaturated substrate is the typical requirement for generating hyperpolarization in hPHIP (Figure 1), meaning that both hydrogen atoms must end up in the same product molecule and their spins remain correlated. This implies that hydrogens must follow each other throughout all catalytic steps. However, this requirement can be lifted when the pairwise addition of pH_2_ to an active catalytic center leads to the formation of a dihydride intermediate where the chemical shift difference between the hydride ligands is small, and a strongly coupled spin pair therefore results. Then, if the lifetime of this intermediate dihydride complex is sufficient, the initial singlet spin order of the pH_2_-derived hydrogen pair (eq 1) can evolve into individual single-spin net polarizations (∓*Î*_*z*_ and ∓*Ŝ*_*z*_, Zeeman orders) of the hydrogen atoms that will consequently be transferred into the final reaction products. In such cases, the hyperpolarization is associated with each of the two pH_2_-derived protons separately, without requiring their spin correlation. Therefore, the initial pH_2_ pair can be separated in subsequent steps of the catalytic cycle, while the hyperpolarization observed in the final product can be derived from only one hydrogen of the pair. This effect is referred to as the oneH-PHIP effect.

**Figure 6:**
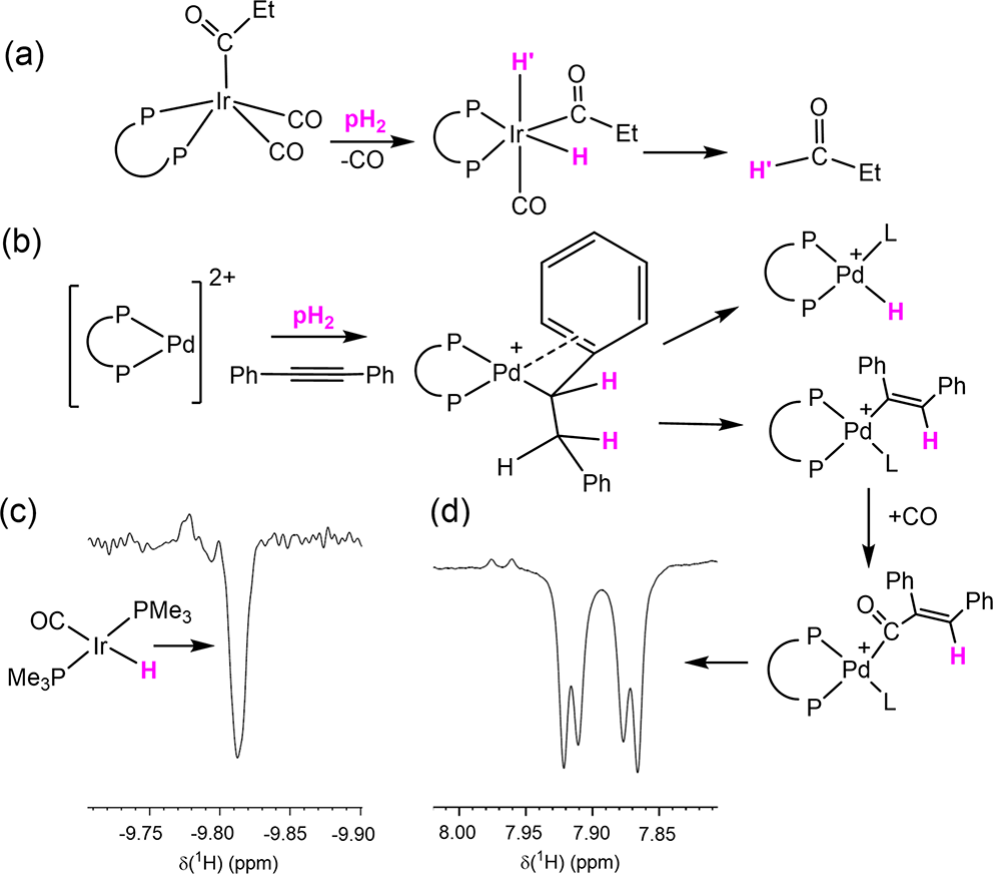
The oneH-PHIP effect has allowed the detection of several single-spin hyperpolarized products. This effect results from the creation of a hyperpolarized AB-type spin system for the pH_2_-derived protons (indicated with pink H atoms in (a) and (b)), with subsequent further reaction producing single spin hyperpolarized products. Examples shown here include (a) aldehydes,^107^ (b) and (c) metal hydride complexes,^154,155^ and (d) vinyl-containing species.^156^ The NMR traces illustrate the typical appearance of a single spin-polarized resonance under ^31^P decoupling in the case of (c).

OneH-PHIP was first observed by Permin and Eisenberg during their stoichiometric studies of hydroformylation by platinum–tin and iridium carbonyl species.^107^ Here, *trans*-PtCl(COEt)(PPh_3_)_2_ proved to react with SnCl_2_ and pH_2_ to form propanal, where only the slowly relaxing aldehydic proton (Figure 6a) exhibited NMR signal enhancement. This is reflective of the creation of single-spin net polarization associated with an *Î*_*z*_ type term (indicated by **H** in the following products), which differs significantly from the more usual pH_2_-derived longitudinal two-spin order *Î*_*z*_*Ŝ*_*z*_ term (the last term in eq 1), which is destroyed when the coupling between the spins is lost. As this section will illustrate, such effects are relatively common, although the signal enhancements are relatively low. For example, a signal enhancement of 5-fold at 9.4 T using 50% pH_2_ was achieved for the aldehydic proton of propanal.^107^ Hence, oneH-PHIP observation has been limited to slowly relaxing species or high-turnover catalysis.

Permin and Eisenberg investigated the catalytic production of propanal (CH_3_CH_2_C**H**O) using PtCl_2_(CO)(PPh_3_)–SnCl_2_^107^ and [Ir(COEt)(CO)_2_(dppe)] (dppe is 1,2-bis(diphenylphosphino)ethane) wherein only the aldehydic proton was hyperpolarized (Figure 6a). In the case of Ir systems, hyperpolarized hydride ligand signals were also detected for the intermediate [Ir**(H)**_**2**_(COEt)(CO)(dppe)], where the hydride ligand *cis* to the phosphines (**H′** in the figure) was then found to become the hyperpolarized aldehydic proton in the final product. Hydride signals for this intermediate appear at very close resonances (−8.696 and −8.905 ppm in benzene), forming an AB-type spin system. In Pople notation, the AB-type spin system corresponds to two spins exhibiting a chemical shift difference comparable to their spin–spin coupling. The observed hyperpolarization of only a single proton in the final aldehyde product was called oneH-PHIP.

Subsequent solvent variation of a benzene–acetone mixture led to an inversion in the oneH-PHIP phase of the aldehydic proton as a consequence of the relative change in chemical shifts of the AB spin system of the hydride ligands inverting. Hence, the oneH-PHIP effect was linked to strong coupling. In this case, a rigorous theoretical description, enunciating the role of chemical shift difference and mutual spin–spin interaction in the AB spin system that created the *Î*_*z*_–*Ŝ_z_* type magnetization was reported, which set Bargon’s earlier description into a firm chemical context.^28^ However, it does not exclude the possibility of a relaxation-driven polarization transfer mechanism. For instance, in section 2.5, we discuss such mechanisms in the hydrogenation of alkynes and imines with pH_2_ using *ansa*-aminoborane catalysts.^148−150^ To verify the exact mechanism, one can study the magnetic field dependence of the oneH-PHIP effect: cross-correlated relaxation is expected to be stronger at higher fields, while coherent mechanisms dominate at lower fields.^28,151,152^ Another rationale for the oneH-PHIP effect appears in ref (153), although this time in the context of the hyperpolarization of water and alcohols by either homogeneous or heterogeneous catalysis, as described in section 2.4.

An additional example of a hydride resonance exhibiting oneH-PHIP was observed during catalytic studies of alkyne hydrogenation by [Pd(bcope)(OTf)_2_] (bcope = (*c*-C_8_H_14_-1,5)PCH_2_CH_2_P(*c*-C_8_H_14_-1,5); OTf = CF_3_SO_2_O^–^) where species like [Pd(bcope)(pyridine)(**H**)](OTf) were detected.^155^ This study extended into the detection of critical reaction intermediates like [Pd(bcope)(CPh=C(**H**)Ph)(pyridine)](OTf), where the single vinyl proton exhibited strong hyperpolarization, alongside *cis*-Ph**H**=C**H**Ph (Figure 6b). During these studies, it was the strongly coupled spin system of the reversibly formed intermediate [Pd(bcope)(CHPhC(H)_2_Ph)](OTf) that led to this behavior.^155^ The related complex, the alkene insertion product, [Pd(Ph_2_PCH_2_CH_2_PCy_2_)(—C(Ph)H—CHPh—CPh=(C**H**)Ph)]OTf has also been observed thanks to the ^1^H NMR signal enhancement of oneH-PHIP^157^ and vinyl ethers have been produced during platinum-catalyzed reactions that exhibit this effect.^158^

Furthermore, the addition of CO to drive palladium-catalyzed carbonylation has extended the hyperpolarized observations to include the ketone MeOCO(CPh)=C**H**Ph proton resonance, alongside further signals in the acyl bearing reaction intermediate [Pd(bcope)(CO—CPh=C(**H**)Ph)(CO)](OTf), the novel hydride complex [Pd(bcope)(CO)(**H**)](OTf), the alkene complex [Pd(bcope)(C**H**Ph=CPh(COOMe)] and free **H**D (alongside **H**_2_) (Figure 6d).^156^ Here again, detecting a hyperpolarized response for the released H_2_ reflects the oneH-PHIP that results from strong coupling effects in species that led to it.

Later, Guan et al. reported on studies of related [Ir(η^3^-C_3_H_5_)(CO)(PMe_3_)_2_] type species and described how the hydride ligand signal for [**H**IrI(CO)(PMe_3_)_2_] exhibited oneH-PHIP (Figure 6c).^154^

It should be apparent from these discussions that the sharing of hyperpolarization between species can occur via numerous processes with dramatically different efficiencies. The polarization of a single proton was also reported in other reactions with H_2_ involving metal-free catalysts or heterogeneous catalysts and led, among others, to a polarization of water, as discussed in the following sections.

### 2.4 SWAMP and NEPTUN

Hyperpolarized water is an important target molecule as it can be used for angiography and perfusion biomedical imaging^159−161^ and as a polarization source for heteronuclear signal enhancement in biomolecular NMR spectroscopy.^10,162−166^ While high polarization levels (>60%) have been shown to result from dDNP,^167^ pH_2_-based methods offer an alternate route that is both rapid and less expensive, thus making the approach more widely accessible.

**Figure 7:**
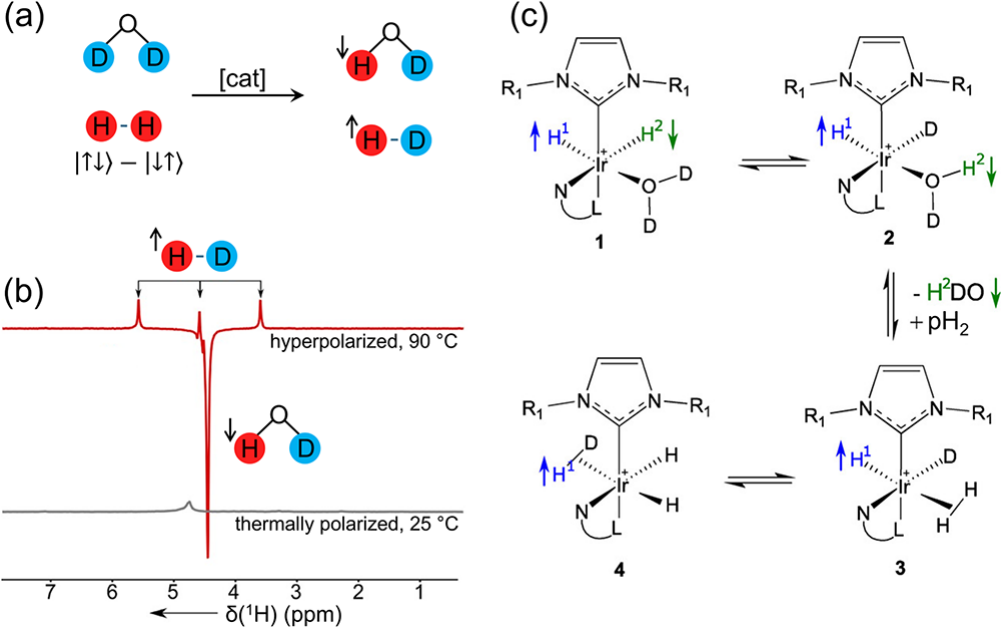
(a) H/D exchange, in the presence of a water-soluble iridium catalyst and histidine, leading to hyperpolarization of HD and HDO and (b) the resulting NMR spectrum, in comparison to the thermally polarized spectrum. (c) The proposed “NEPTUN” mechanism underpins the spectrum. *(a, b) Adapted with permission from Lehmkuhl et al*.^168^*Copyright 2017 John Wiley and Sons. (c) Adapted with permission from Emondts et al*.^109^*Copyright 2018 John Wiley and Sons*.

Water had been an elusive target for PHIP until 2017, when it was hyperpolarized in D_2_O mixtures of l-histidine and a water-soluble iridium complex, [Ir(Cl)(IDEG)(COD)] (IDEG = 1,3-bis(3,4,5-tris(diethylene glycol)benzyl)imidazole-2-ylidene).^168^ In this system, the hyperpolarized HDO and HD proton signals appear in the emission and absorption phases, respectively (Figure 7a,b). Consistent with the oneH-PHIP theory (see section 2.3),^109,153^ the enhanced HD and HDO signals have opposite phases and very similar field dependencies of their signal amplitudes, which reach a maximum near 45 mT where the *J*-coupling and chemical shift difference between the dihydride protons (Figure 7c) are matched. The corresponding oneH-PHIP mechanism, mediated by (*i*) H/D exchange with coordinated D_2_O, (*ii*) dissociation of HDO, and (*iii*) H–D recombination, was named nuclear exchange polarization by transposing unattached nuclei (NEPTUN).^109^ The role of l-histidine in this work remains unclear.

A NEPTUN-type mechanism was also suspected to be active in relayed hyperpolarization experiments where the goal was to further extend the SABRE hyperpolarization to heteronuclei in noncoordinating substrates like alcohols (e.g., methanol, ethanol) via proton exchange with a carrier amine.^169^ The possible involvement of such a mechanism, in addition to OH/NH exchange, was inferred from magnetic field dependencies of ^13^C distortionless enhancement by polarization transfer (DEPT) signals, which, in addition to showing a peak at 6.5 mT, as expected for the conventional SABRE matching condition (relayed to the target via NH/OH exchange), there is an even more prominent peak after transfer at 19.2 mT, which is hypothesized to stem from the NEPTUN effect.^169^ However, attempts to observe the hydride resonances indicative of NEPTUN directly were unsuccessful.

While most PHIP studies utilize dissolved organometallic catalysts, heterogeneous catalysis offers facile separation of the hyperpolarized products from the catalyst and can even be used in a packed-bed flow-reactor configuration.^170,171^ Supported noble metals are among the most active hydrogenation catalysts. Unfortunately, only a tiny fraction of adducts (ca. 1–5%) are formed by pairwise addition, depending on nanoparticle size and reaction conditions. Rapid H adatom diffusion and facile exchange with gaseous H_2_ conspire to destroy the singlet order in the nascent H adatom pair.^15,50,172,173^ Intermetallic phases incorporating an active and inactive metal, such as Pt and Sn, allow for the tuning of molecular adsorption and diffusion dynamics through a combination of geometric and electronic effects.^174−176^ Thus, as the fraction of Sn increases across the series Pt → Pt_3_Sn → PtSn, the pairwise selectivity for hydrogenation of propene increases by more than 3 orders of magnitude.^174^ After bubbling pH_2_ through a D_2_O suspension of Pt_3_Sn@mSiO_2_ (Pt_3_Sn nanoparticles encapsulated in mesoporous silica) for 30 s, Zhao et al. observed hyperpolarization of the residual protons of solvent molecules.^153^ The effect was dubbed surface waters are magnetized from parahydrogen (SWAMP). Proton hyperpolarization in methanol-*d*_4_ and ethanol-*d*_6_ was also observed. The surface properties of Pt_3_Sn now balance the necessary facile H_2_ activation and suppression of diffusion.

**Figure 8:**
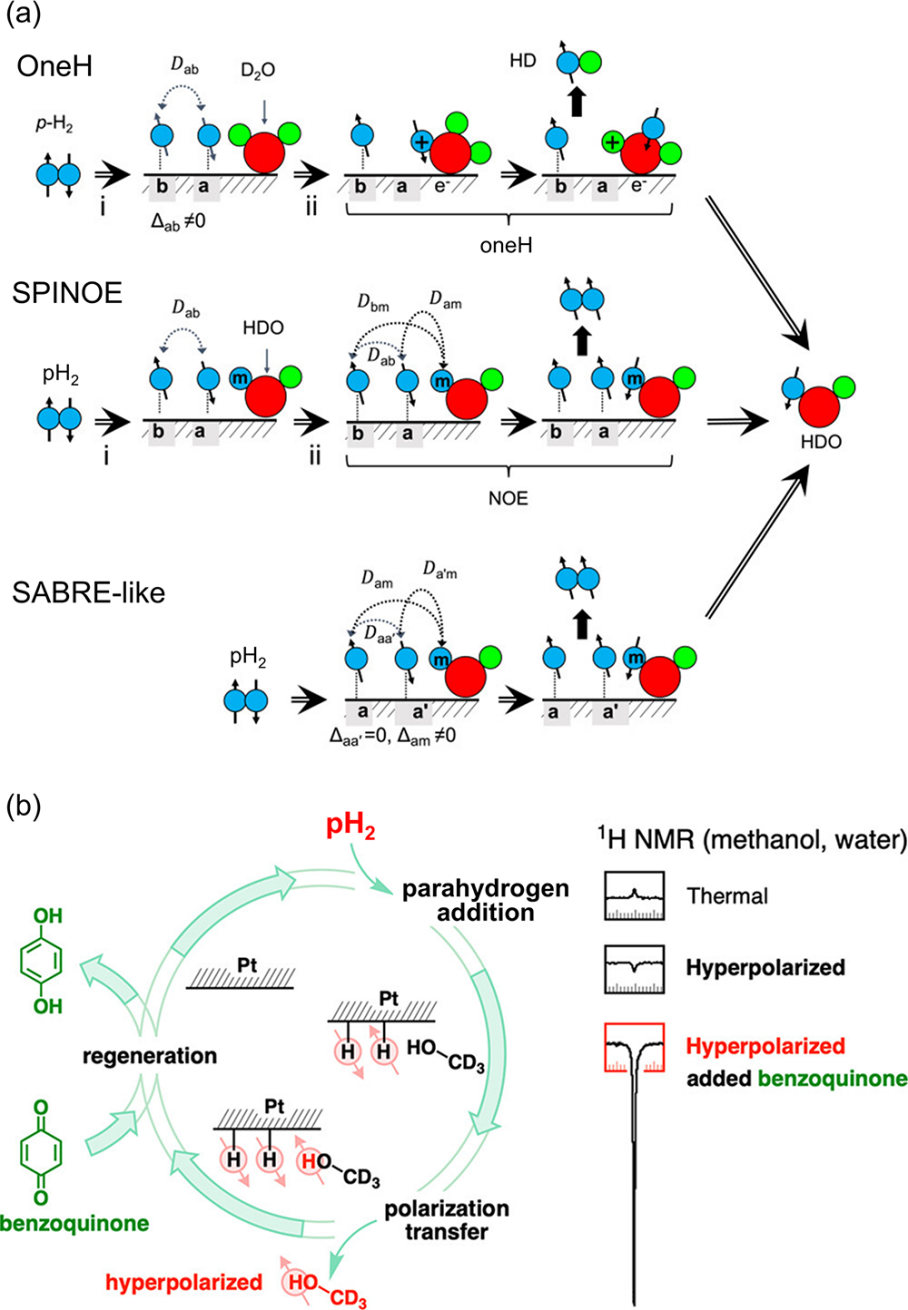
(a) Possible mechanisms underpinning surface-mediated hyperpolarization of liquid water. (b) Mechanism of benzoquinone scavenging of depolarized H adatoms on Pt/C, and its effect on the SWAMP signals. *(a) Adapted with permission from Zhao et al*.^153^*Copyright 2018 Elsevier. (b) Reproduced from Norcott*.^178^*Copyright 2023 American Chemical Society. This publication is licensed under CC-BY-NC-ND 4.0*.

The NEPTUN and SWAMP systems share a few similarities: (i) emission phase of the HDO peak. (ii) absence of signal enhancement at zero or high magnetic field, revealing a role of Zeeman interactions; (iii) monotonic growth of [HDO] with total pH_2_ bubbling time; (iv) emergence of a dissolved HD (triplet) NMR signal. The last two are accounted for by the net isotope exchange reaction described in eq 3.
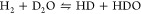
3For Pt surfaces, H/D exchange is mediated by reversible electron transfer from an H adatom to the metal and proton transfer to surface water to yield a hydronium-like species where H/D exchange occurs.^177^ However, the oneH-PHIP/NEPTUN mechanism was only tentatively excluded by preliminary data revealing the different dependences of the SWAMP signals for exchangeable and nonexchangeable protons of HOCD_3_ and DOCHD_2_, respectively, on the total amount of H/D exchange. Plausible mechanisms are illustrated in Figure 8a.

While the monometallic Pt@mSiO_2_ nanoparticles of ref (153) were found to be inactive as SWAMP catalysts, Norcott discovered that one could hyperpolarize water and methanol using a commercially available carbon-supported Pt nanoparticle catalyst when benzoquinone was added to a D_2_O suspension of the catalyst.^178^ Maximum ^1^H NMR signal enhancements (relative to thermal equilibrium at 1.4 T) approaching 45-fold were observed for methanol with ten equivalents of benzoquinone (w/w with respect to Pt/C). Benzoquinone is converted to hydroquinone during this process, which assists in increasing the turnover of fresh pH_2_ on the surface and thereby increasing the level of polarization of methanol and water (Figure 8b).

While such signal enhancements are likely to increase with further catalyst development and optimization of experimental conditions, it remains to be seen whether the PHIP approach can rival the very high polarization levels achievable by dDNP for water.^159,160,162−165^ As pH_2_-based hyperpolarization techniques are inherently rapid, continuous, and low-cost, their use to achieve sufficient levels of water hyperpolarization could provide advantages to dDNP methods and significantly advance the application range of hyperpolarized water in biomedical research.

### 2.5 Metal-Free PHIP: Molecular Tweezers and pH_2_ Activators

**Figure 9:**
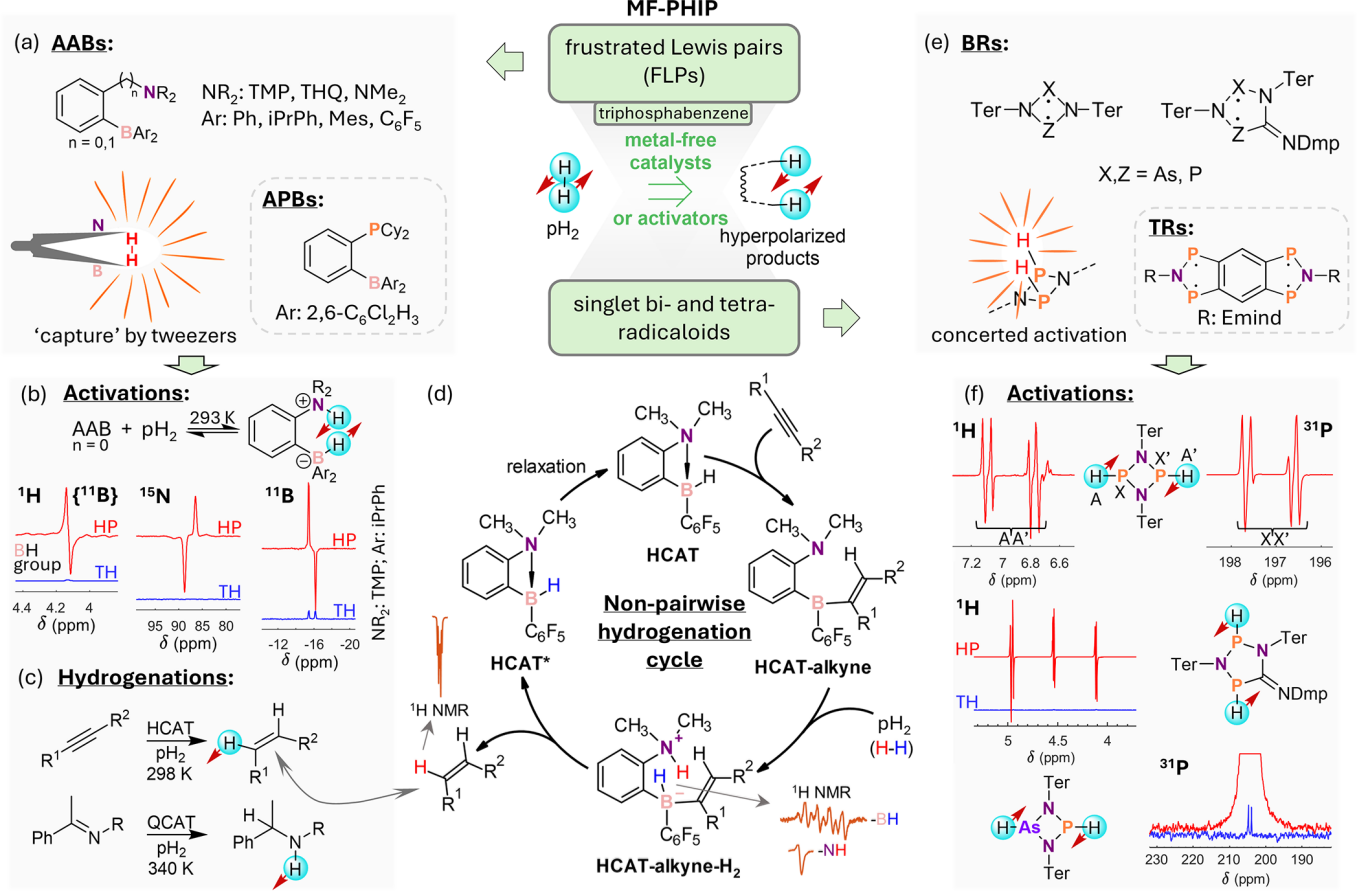
An overview of metal-free PHIP (MF-PHIP). The central top part highlights that MF-PHIP effects have been demonstrated in activation of pH_2_ using frustrated Lewis pairs (FLPs) and using bi- and tetraradicaloids (BRs and TRs). Correspondingly, the structures of *ansa*-aminoborane (AAB) and *ansa*-phosphinoborane (APB) FLPs are presented in (a). AABs are referred to as molecular tweezers for pH_2_. pH_2_ activation under ambient conditions using *ortho*-phenylene AABs (*n* = 0) accompanied by the corresponding hyperpolarized NMR spectra is illustrated in (b). See (a) for the definition of n. In addition to ^1^H, ^15^N and ^11^B nuclei are also hyperpolarized spontaneously at high magnetic fields in this process. Alkyne and imine hydrogenation reactions catalyzed by AABs HCAT (*n* = 0; NR_2_ = NMe_2_; Ar = C_6_F_5_; R′ = H) and QCAT (*n* = 1; NR_2_ = THQ; Ar = C_6_F_5_), respectively, are shown in (c). Additionally, the catalytic cycle of alkyne hydrogenation using the HCAT AAB catalyst is presented in (d). Typical ^1^H NMR signals of the reaction intermediate and the reaction product are shown next to the corresponding structures in the cycle. Structures of BR and TR molecules that demonstrated hyperpolarization effects in pH_2_ activations are depicted in (e). Corresponding examples of enhanced ^1^H and ^31^P NMR signals observed in reactions with BR molecules are shown in (f). The structures of the corresponding BR–H_2_ adducts are depicted next to the spectra. Abbreviations: TMP = N-2,2,6,6-tetramethylpiperidinyl; THQ = N-tetrahydroquinolinyl; iPrPh = 2-isopropylphenyl; Mes = mesityl; Cy = cyclohexyl; Ter = 2,6-dimesitylphenyl; Dmp = 2,6-dimethylphenyl; Emind = 1,1,7,7-tetraethyl-3,3,5,5-tetramethyl-s-hydrindacenyl. *(d) Adapted from Zakharov et al*.^148^*Copyright 2022 John Wiley and Sons. This publication is licensed under CC-BY 3.0*.

The chemical activation of pH_2_ is crucial to derive enhanced NMR signals in PHIP. Commonly, transition metal catalysts are employed to mediate such activations and produce hyperpolarized substances. At the same time, the use of metal-free activators and catalysts for pH_2_-based hyperpolarization, collectively named metal-free PHIP (MF-PHIP), is also documented.^129,148,149,179−186^ This section focuses on several types of MF catalysts, their structures, hyperpolarization effects, and their unique mechanistic features (Figure 9).

MF activations of H_2_ are less common than those that rely on transition metal centers. They have recently attracted a lot of attention due to the possibility of using sustainable main-group elements to design less toxic and more environmentally friendly catalysts. MF catalysts for PHIP is an emerging research field that is still in its infancy. In this regard, frustrated Lewis pairs (FLPs)^187^ are the most studied class of MF activators for pH_2_. Specifically, various *ansa*-aminoborane (AAB) FLPs show pronounced hyperpolarization effects (Figure 9a). These compounds are referred to in the literature as “molecular tweezers” that stretch but do not split H_2_ molecules.^188^ Recent studies revealed that the stretched H–H bond is relatively weak, making it possible to form various rotomeric forms in solution, including those with large H···H separations.^183^ Nevertheless, AAB–H_2_ adducts have motionally averaged *J*-coupIing constants (2–4 Hz) between the ^1^H–^1^H pair, allowing for PHIP effects under high field (PASADENA) conditions.^129,183^

Unlike homolytic oxidative addition to metal centers, AABs activate pH_2_ heterolytically with a clear charge separation on the Lewis acidic boron and the Lewis basic nitrogen sites (Figure 9b), although the two protons remain spin correlated. Depending on the AAB structure, hyperpolarization of ^1^H, ^11^B, and ^15^N can be observed in simple pH_2_ bubbling experiments without harnessing dedicated pulse sequences.^36^ Signal enhancements as large as 2000-fold at 9.4 T and room temperature have been observed in the resulting ^1^H NMR spectra for the pH_2_-originating protons of AAB–H_2_. The size of this ^1^H NMR signal gain depends strongly on the experimental conditions and is defined by relaxation and kinetic parameters.^183^ Density functional theory (DFT) calculations have also revealed various conformational forms of AAB adducts and their transformations.

In addition to simple pH_2_ activation, *ansa*-aminoboranes can be used in catalytic hydrogenations of alkynes^148^ and imines^149^ with pH_2_ (Figure 9c). Interestingly, the catalytic cycles that lead to hyperpolarized akenes and amines are nonpairwise, implying that the pH_2_-derived protons end up in different product molecules (Figure 9d). The hyperpolarization effects in this case are not expected to be observable in PHIP. However, due to the strong chemical shift anisotropy (CSA) of NH protons in the catalytic intermediates, a net negative polarization is generated from the pH_2_ spin order through CSA–dipole–dipole cross-correlated relaxation.^149^ For instance, in alkyne hydrogenations, this mechanism is revealed by the negative in-phase resonance of the NH group of HCAT–alkyne–H_2_ intermediate. As this proton transfers to the final alkene product, a two-orders-of-magnitude enhanced negative signal of one of the added protons at the double bond of the resulting alkene appears in the ^1^H NMR spectra at 9.4 T. This effect is related to oneH-PHIP in hydroformylation reactions catalyzed by metals (section 2.3), but the underlying mechanisms of hyperpolarization, as well as chemical processes, are different. It is worth noting that the ability of cross-correlated relaxation to transform pH_2_ spin order to a net polarization was also observed on metal complexes, e.g., by Aime et al. in pH_2_ activations using Os and Ru clusters.^189,190^

AABs are reported to be generally water-intolerant, which is a significant obstacle to the wide application of MF-PHIP. Valuable steps have been made to resolve this issue recently.^267^ In addition, *ansa*-phosphinoboranes (APBs) were demonstrated to show PHIP effects in the presence of several equivalents of H_2_O (Figure 9a).^182^ Other FLPs showing hyperpolarization effects include Sn/P systems,^191^ though it is strictly not a MF compound and will not be discussed here. In addition, aromatic triphosphabenzene was also shown to reveal hyperpolarization in the reaction with pH_2_ at elevated temperatures (375 K).^179^ However, the resulting H_2_ adduct is prone to decomposition.

Singlet pnictogen radicaloids are another class of MF pH_2_ activators that demonstrate prominent hyperpolarization effects. Electron spins in these open-shell molecules are coupled into a singlet state that does not possess free electron angular momentum, which excludes the deleterious influence of the radical centers on the nuclear spins. Typical examples include cyclic species with P and/or As radical centers isolated by surrounding bulky substituents for stabilization. This configuration maintains high reactivity toward small molecules, such as H_2_, while preserving an open-shell structure. In the context of PHIP, biradicaloids (BRs) with four- or five-membered cycles are studied more extensively (Figure 9e), and any observed hyperpolarization effects strongly depend on BR symmetry.^185^ With symmetric four-membered biradicaloids, pH_2_ forms symmetric adducts. For instance, four-membered baricaloids form the AA′XX′ spin system, which leads to enhanced ^1^H and ^31^P NMR signals in PASADENA experiments (Figure 9f).^184,185^ Nonsymmetric five-membered species form a system with weakly coupled protons, leading to only ^1^H hyperpolarization. However, the transfer of ^1^H hyperpolarization to ^31^P can be achieved using ESOTHERIC NMR pulse sequences with ^31^P NMR signal enhancements exceeding 3 orders of magnitude at 9.4 T.^185,192^ Tetraradicaloids (TRs) are represented by a single example (Figure 9e),^186^ which showed less pronounced but interesting hyperpolarization for the addition of the first and second equivalents of pH_2_. Radicaloid systems are generally more reactive than FLPs, which makes them especially interesting for future developments that may lead to active catalysts, e.g., for hydrogenation or hydroformylation reactions.

MF-PHIP represents an exciting frontier in hyperpolarization method development. Using MF compounds such as FLPs and radicaloids introduces new mechanistic pathways and structural features that differentiate them from traditional metal-catalyzed systems. The unique hyperpolarization effects and mechanisms of MF-PHIP, including two-centered activation and nonpairwise hydrogen transfer, offer potential for innovative applications. Future research in this area promises to further enrich our understanding and utilization of these novel catalysts for hyperpolarization.

### 2.6 Revealing PHIP in Subsequent Chemical Transformations

The involvement of hyperpolarized molecules in subsequent chemical transformations is an interesting application of PHIP that is nicely illustrated using metabolic reactions, such as pyruvate-to-lactate conversion^193^ and fumarate-to-malate^194^ conversions. Other examples include oxidation of hyperpolarized pyruvate with H_2_O_2_^195^ or decarboxylation with yttrium polyaminoacarboxylate adducts,^196^ methylation of N-heterocycles,^197^ and conversion of hyperpolarized ^15^NO_2_^–^ via several reactions to make a range of products with enhanced ^15^N NMR signals,^198^ and other transformations.^199^

**Figure 10:**
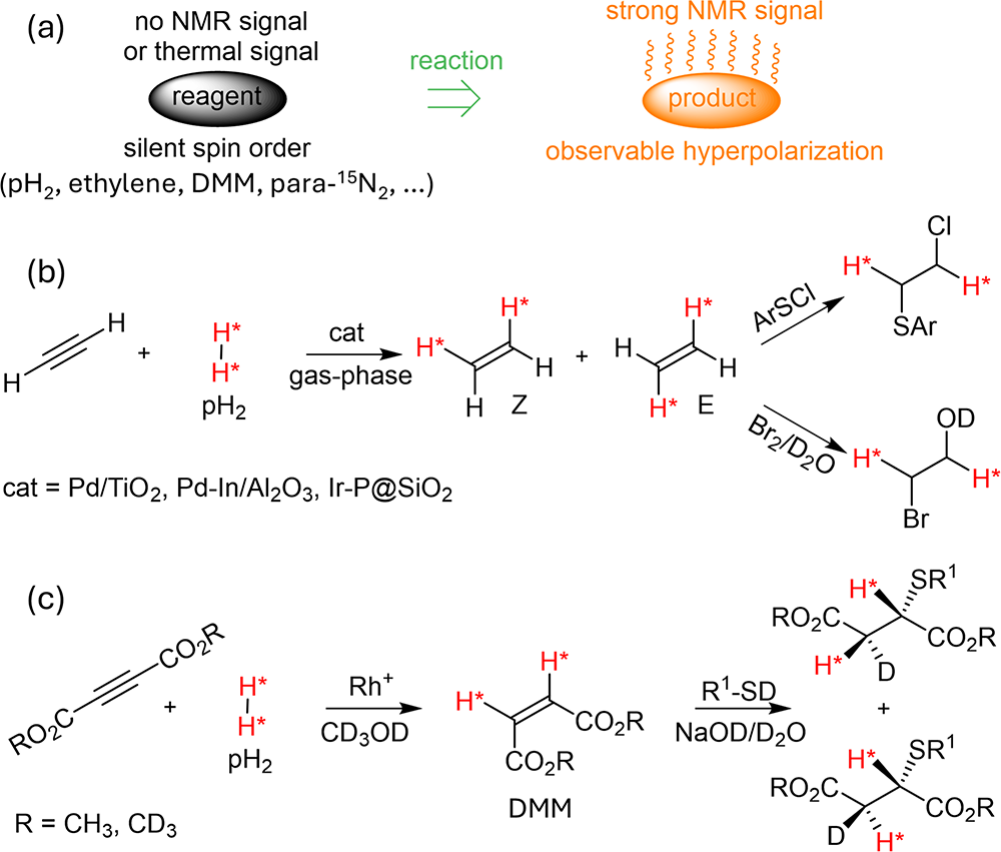
(a) A general scheme of formation of compounds with hyperpolarized spins in chemical reactions of symmetric molecules, such as pH_2_, ethylene, DMM, and para-^15^N_2_, accommodating otherwise unobservable nuclear spin orders. (b) The chemical synthesis of *Z*- and *E*-ethylene from acetylene and pH_2_ over various heterogeneous catalysts, as well as subsequent reactions revealing hyperpolarization. (c) The chemical synthesis of dimethyl maleate (DMM) molecules in hydrogenation with pH_2_ over a cationic Rh^+^ catalyst, followed by their subsequent reaction with thiol molecules to reveal hyperpolarization. Abbreviations: DMM - dimethyl maleate.

Typically, PHIP-enhanced NMR signals are visible without any subsequent reaction and can serve as a tool for kinetic measurements and identifying intermediates or additional products of the following reactions. However, if we consider symmetric molecules produced in reactions with pH_2_, the product, although in a far from thermodynamic equilibrium nuclear spin state, may not exhibit observable hyperpolarization if the pH_2_ addition site is at the center of symmetry. In this case, a subsequent symmetry-breaking chemical reaction(s) may be required to reveal otherwise unobservable hyperpolarization (Figure 10a). The quintessential example of this kind is the pH_2_ molecule, which accommodates singlet nuclear spin order and does not yield NMR signals. As first shown by Bowers and Weitekamp,^17,18^ one needs to break the symmetry of pH_2_ in a chemical reaction to observe hyperpolarization. Notably, symmetric molecules can typically host long-lived spin orders.^200,201^ Such molecules can be synthesized using pH_2_ in hydrogenative and non-hydrogenative reactions. For example, the syntheses of ethylene from acetylene and pH_2_ (Figure 10b), dimethyl maleate (DMM) from dimethyl acetylene dicarboxylate (DMAD) (Figure 10c), and para-^15^N_2_ using SABRE were described.^202−209^

The case of ethylene is fundamentally interesting since it, like H_2_, has nuclear spin isomers of molecules (NSIMs) that differ by rotational and spin degrees of freedom due to the coupling of nuclear spin and rotational states through the symmetry properties of their respective wave functions.^210^ Briefly, there are four NSIMs for ethylene that can be classified according to the symmetries of the nuclear spin state for the ***D***_2h_ molecular point group using Mulliken symbols: A_g_ (one quintet and two singlets), B_1u_ (triplet), B_2u_ (triplet), and B_3g_ (triplet). Depending on the stereoselectivity of the hydrogenation of acetylene, *syn* and *anti* pH_2_ addition products, *Z*- and *E*-ethylene, respectively, can be produced (Figure 10c),^202^ which is primarily determined by the hydrogenation catalyst employed. For instance, supported Pd nanoparticles are less selective and produce both *Z*- and *E*-ethylene products,^202^ whereas immobilized complexes of Ir are more selective, leading primarily to *Z*-ethylene.^205^ Interestingly, the subsequent reactions of ethylene produced using different catalysts with sulfenyl chlorides^202^ or Br_2_/D_2_O^205,211^ reveal different lifetimes of the nonequilibrium spin states in ethylene. This was rationalized based on the interconversion between different NSIMs of ethylene,^204^ providing insights that in the gas phase, *Z*-ethylene has only one long-lived component. In contrast, *E*-ethylene has two long-lived components due to the imbalance of NSIMs with different inversion symmetry in the latter case.^202,205^ Lifetime constants of more than 15 min were measured by unlocking the hyperpolarization in the subsequent reactions for gaseous *E*-ethylene.

Another example of revealing the latent polarization inherited from pH_2_ is documented for the reaction of thiols with DMM produced from DMAD in a liquid-state hydrogenation over a Rh(I) cationic catalyst (Figure 10c).^203^ As in the case of ethylene, storage of the nonequilibrium nuclear spin order after the hydrogenation was demonstrated in this study. The thiol reaction allowed for the lifetime measurement of the populated long-lived singlet spin order at a high field of up to 4.7 min. Interestingly, the unique symmetry of the DMM molecule, which induces slight magnetic inequivalence in the added pH_2_-derived proton pair, allows alternative methods that do not require chemical transformation to reveal hidden singlet spin state populations. Instead, such hidden spin states can be converted into observable magnetization using magnetic field cycling^212^ or applying RF fields.^213−215^ These methods do not apply to ethylene, as all its protons are magnetically equivalent.

In addition to ethylene and DMM, para-^15^N_2_ has also been reported to form from SABRE-hyperpolarized ^15^N-labeled tetrazine^208^ and diazirines^209^ in chemical reactions involving these agents. However, the successful formation of para-^15^N_2_ in these cases was inferred only from the absence of a ^15^N_2_ signal in ^15^N NMR spectra after its production step. Similarly to pH_2_, para-^15^N_2_ is NMR silent. So far, no subsequent symmetry-breaking reaction of para-^15^N_2_ that reveals its singlet spin order has been reported to our knowledge.

Overall, the availability of methods that use pH_2_ to produce symmetric molecules with long-lived nuclear spin orders can expand the range of chemical reactions studied by providing flexible time windows. For example, it can enable one to go beyond conventional hydrogenation studies using pH_2_ in PHIP, extending to electrophilic additions to double bonds, as demonstrated in the cases of ethylene and DMM. Furthermore, the analysis of the generated spin order lifetimes in molecules such as ethylene and para-^15^N_2_ can provide essential insights into the fundamentals of NSIMs and underlying molecular physics.

### 2.7 Spreading Hyperpolarization via Chemical Exchange: PHIP-X and SABRE-RELAY

**Figure 11:**
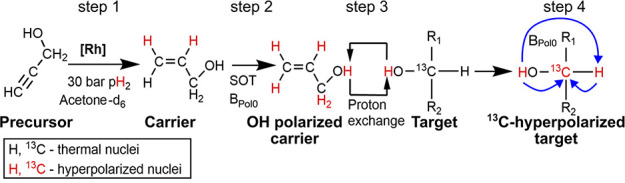
Schematic view of parahydrogen-induced polarization by chemical exchange (PHIP-X). PHIP-X consists of four essential steps: hydrogenation of the carrier agent (step 1), the polarization of the exchanging protons (step 2), transfer of the exchanging protons from the carrier to the target molecule (step 3), and polarization of the target nucleus (step 4) using RF-induced spin order transfer technique or free evolution at low and ultralow magnetic fields. *Adapted from Them et al*.^219^*Copyright 2024 Springer Nature. This publication is licensed under CC BY 4.0*.

**Figure 12:**
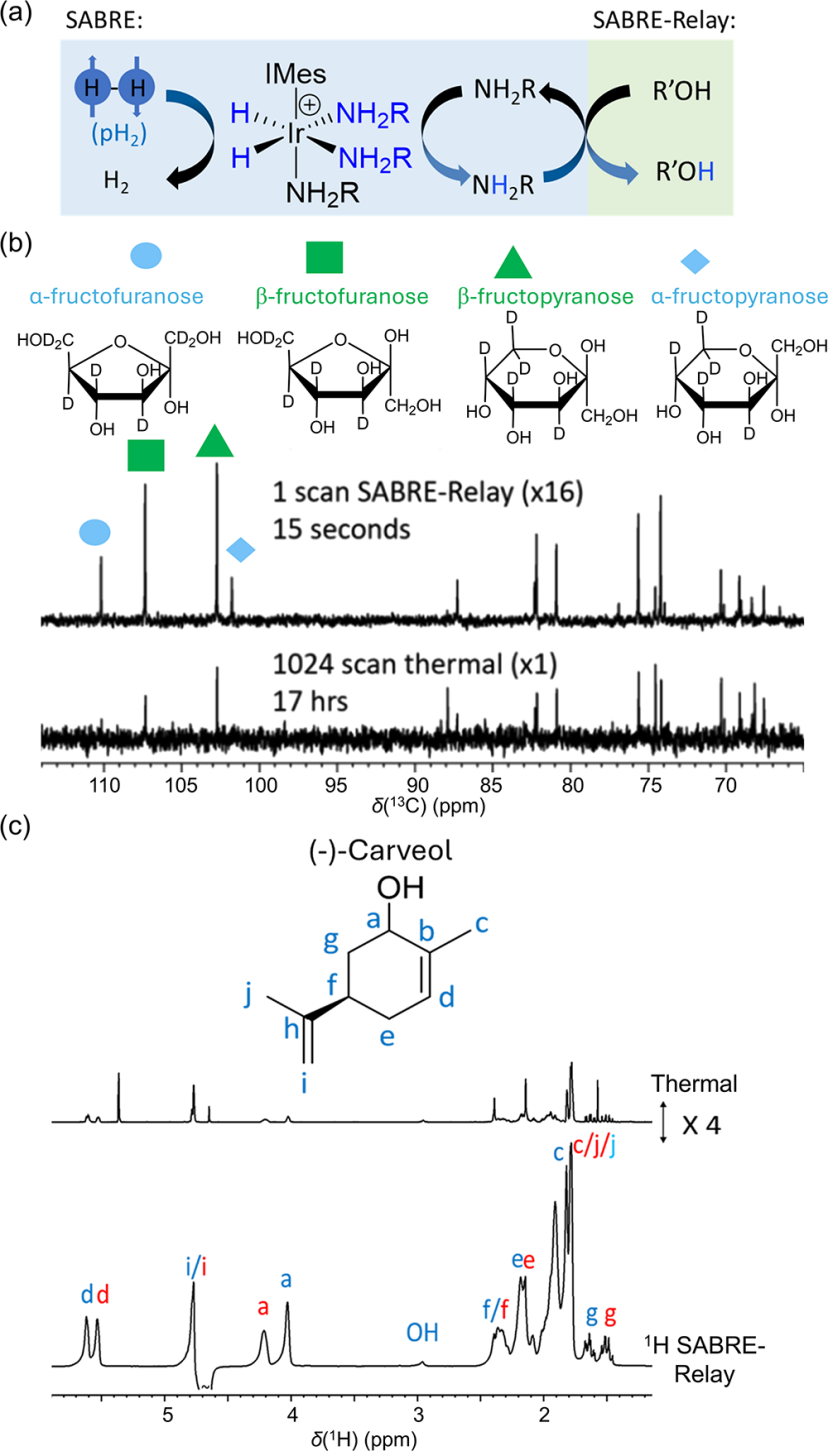
Demonstration of the SABRE-Relay effect. (a) Depiction of the SABRE-Relay method. (b) SABRE-Relay can allow quantification of isomer ratios of fructose in a single scan ^13^C measurement or (c) quantification of diastereomer ratios of the natural product (−)-carveol from a single scan ^1^H measurement. *Details for (b)*: ^13^C{^1^H} NMR spectra (right) acquired for 40 mM of d-fructose (natural ^13^C abundance) with 23.8 mM benzyl-*d*_7_-amine and 4.8 mM of [Ir(Cl)(COD)(SIMes-*d*_22_)] (where COD is *cis*,*cis*-cyclooctadiene and SIMes is 1,3-bis(2,4,6-trimethylphenyl)-4,5-dihydroimidazol-2-ylidene) in a 0.65 mL DCM-*d*_2_:DMF (1.6:1) mixture measured at 9.4 T. The bottom spectrum shows the result of a thermally polarized signal averaging over 1024 scans (approximately 17 h), and the middle spectrum represents the single scan SABRE-Relay hyperpolarization measurement recorded after shaking the sample with pH_2_ at 6.5 mT. *Details for (c)*: Exemplar single scan thermally polarized (above) and ^1^H SABRE-Relay hyperpolarized (lower) ^1^H NMR spectra for a sample of [IrCl(COD)(IMes)] (5 mM), NH_3_ (30 mM), (−)-carveol (25 mM) and pH_2_ (3 bar) in DCM-*d*_2_ (0.6 mL). The resonance labels in red and blue correspond to the two diastereomers. The hyperpolarized NMR spectrum is recorded immediately after shaking the sample for 10 s with fresh pH_2_ at 6.5 mT. *(a, c) Adapted from Alshehri et al*.^225^*Copyright 2023 Royal Society of Chemistry. This publication is licensed under CC BY 3.0. (b) Adapted from Richardson et al*.^223^*Copyright 2019 Royal Society of Chemistry. This publication is licensed under CC BY 3.0*.

In recent years, a new approach has emerged to boost the NMR signal for molecules that do not contain functionalities suitable for direct PHIP or SABRE (natively or on a side arm). Two versions of this approach are based on reversible proton exchange between a hyperpolarized carrier and a to-be-hyperpolarized molecule and are termed PHIP by chemical exchange (PHIP-X)^216^ and SABRE-Relay.^217^ In both methods, traditional PHIP or SABRE is used to polarize a “transfer” or “carrier” molecule, whose polarization is then transferred to a secondary target molecule via the exchange of hyperpolarized OH/NH protons (Figures 11 and 12a). These approaches have allowed a significant expansion of the substrate scope of PHIP and SABRE in recent years.

In the case of PHIP-X^216^ (also referred to as PHIP-Relay),^218^ propargyl alcohol, propiolic acid, or propargyl amine were hydrogenated with pH_2_ using a homogeneous Rh catalyst in an aprotic solvent, such as acetone, to produce a hyperpolarized “transfer” agent (Figure 11).^216^ Strong spin–spin interactions distribute the polarization among the protons of the transfer agent, including the labile OH (or NH) proton. The polarization of this labile proton is relayed to the spin system of a third molecule by chemical exchange, where, again, spin–spin couplings, low magnetic field, or RF spin order transfer sequences (RF-SOT) facilitate the transfer of the polarization to other nuclei such as ^1^H, ^13^C, or ^15^N.^218,219^ PHIP-X was shown to spontaneously polarize labile protons to ca. 0.4% for ethanol and water, 0.07% for lactic acid, 0.005% for pyruvic acid, and at least 0.009% ^13^C polarization for glucose. Using RF-SOTs, 1.2% ^15^N polarization was achieved for urea, where the ^15^N coupling to the labile proton is large, 0.024% for ^13^C glucose,^218^ 0.026% for ^13^C lactate^219^ and ca. 0.007% for ^13^C methanol.^219^ The balance here is reached when proton exchange is slow enough to allow the *J*-coupling to transfer polarization to the labile proton of the carrier first and then from the labile proton to other nuclei of the target. At the same time, the exchange must be fast enough such that spin relaxation would not destroy polarization before the target is polarized. Therefore, even higher polarization values are expected to be achievable after thoroughly tuning the exchange parameters. Also, the polarization transfer is faster and more efficient when transferred to nuclei directly bound to the labile proton, such as ^15^N or ^13^C, with the strongest *J*-coupling constants. An advantage of using PHIP to hyperpolarize the transfer agent compared to SABRE is that the molecule can be polarized up to unity by the direct addition of pH_2_; a disadvantage is that it is irreversible as the addition step can be performed only once (one addition of pH_2_ per transfer agent).

In SABRE-Relay, classical SABRE is used to hyperpolarize ligating carriers, typically NH_3_ and amines, with secondary NH/OH exchange effectively relaying polarization to nonligating target substrates.^220,221^ Consequently, SABRE-Relay has been used to hyperpolarize alcohols,^169,222^ sugars,^223^ silanols,^78^ lactate esters,^224^ natural products,^225^ and many other functional groups^217,226^ that do not interact with the SABRE catalyst directly. As SABRE-Relay, like PHIP-X, depends on transferring proton magnetization, direct polarization of heteronuclei (analogous to SABRE-SHEATH) is impossible. However, the polarization of the exchanging OH group allows the polarization of heteronuclei either spontaneously (i.e., by free evolution), or with the help of RF-SOT. So far, SABRE-Relay has achieved 2.6% ^1^H,^222^ 2.3% ^29^Si,^78^ 1.1% ^13^C,^222,223^ 0.2% ^19^F,^222^ and 0.04% ^31^P^222^ polarization levels. These NMR signal enhancements are generally lower than typical values achieved by conventional SABRE because the relayed polarization is derived from a finite carrier polarization affected by spin relaxation during the chemical exchange. Current studies have optimized factors such as amine type and concentration ratios to increase the target polarization.^222,224,225^

A major limitation of PHIP-X and SABRE-Relay is that they cannot be performed in alcohol or aqueous solvents as their exchangeable protons will compete with the ones of the target molecule. Accordingly, they are commonly performed in dry dichloromethane or chloroform, which may pose a significant challenge for insoluble substrates in these media. SABRE-Relay has already shown potential in molecular analysis. It can enhance the NMR signals of sugars^223^ and natural products^225^ at concentrations as low as tens of micromolar with a single NMR scan. Notably, it can give single-scan quantification of isomeric ratios for OH-containing molecules, such as α and β forms of glucose and fructose (Figure 12b)^223^ or diastereomers of natural oils like (−)-carveol (Figure 12c).^225^ Thanks to the continuous nature of polarization production in SABRE, relayed polarization transfer is expected to become better understood, improved to increase polarization levels, and applied to an ever-increasing scope of target molecules in the years ahead.

Compared to direct pH_2_ addition (PHIP-X), using reversible exchange (SABRE) to polarize the transfer agent has the advantage that the transfer agent can be continuously repolarized; a disadvantage is that the polarization yield is usually lower. Despite recent progress,^219,224,227^ the detailed and quantitative description of chemical exchange and the spread of polarization within the target is still not fully understood.

### 2.8 Perspectives: PHIP in Enzymatically Catalyzed Reactions

**Figure 13:**
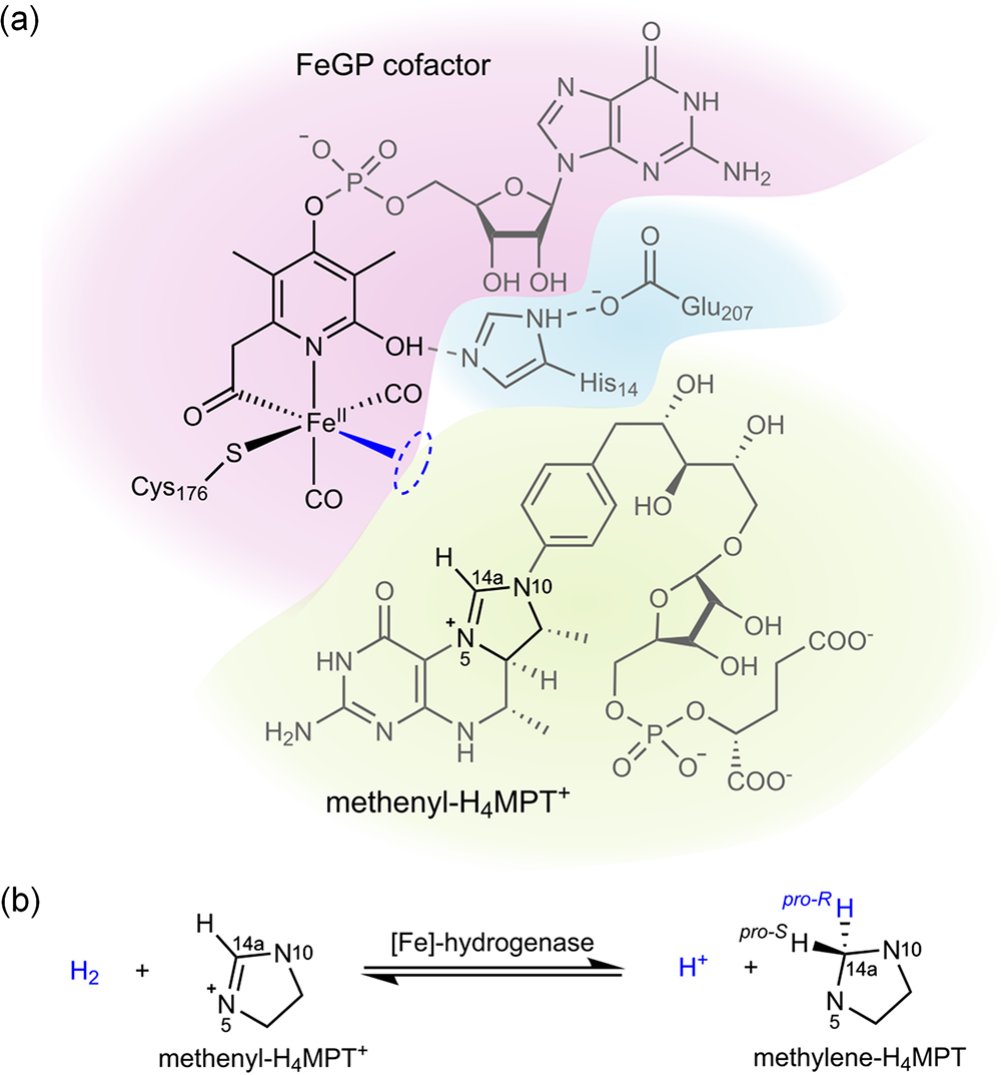
Active site of the mono iron hydrogenase and the hydrogenation reaction. (a) The active site of the [Fe]-hydrogenase, including the iron guanylyl pyridonol and methenyl-H_4_MPT+. (b) Hydride transfer to the H*pro*-R position of the substrate forming methylene–H_4_MPT. *Adapted from Kaltschnee et al*.^151^*Copyright 2024 Springer Nature. This publication is licensed under CC-BY 4.0*.

The use of pH_2_ and hydrogen–deuterium scrambling has been explored in a non-NMR context for several decades to study kinetics and intermediates of enzymes thanks to the slow conversion of pH_2_ to oH_2_ in pure water of about tens to hundreds of minutes.^136^ Such scrambling can thereby help to understand the chemisorption and exchange process by forming HD based on a hydrogen and deuterium source.^228^ Detection of oH_2_ formation after supplying pH_2_ yields information on the splitting and recombination of H_2_.^229^ This information can then give a clearer kinetic picture of hydrogen-activating enzymes.^230^ The particular focus, therefore, is on hydrogenases,^228−231^ an important class of enzymes involved in the hydrogen activation process of, e.g., anaerobic organisms.^232−235^ Hydrogenases promise to be blueprints for eco-friendly catalysts for hydrogen activation on the way to produce green energy.^236^ Therefore, understanding the detailed hydrogen activation of hydrogenases could facilitate the design of potent eco-friendly catalysts. Three different types of hydrogenases are currently known: [FeFe]-hydrogenases, [NiFe]-hydrogenases, and the [Fe]-hydrogenase.^237^ While reaction intermediates of the first two hydrogenases could be well characterized by available methodologies such as EPR, X-ray diffraction, and IR, investigating the [Fe]-hydrogenase has proven to be more challenging. This is because the iron center of the [Fe]-hydrogenase is Fe^II+^ encapsulated in a guanylylpyridinol (FeGP) and remains in a diamagnetic state through the whole catalytic cycle (Figure 13a). Under catalytic conditions, methenyl-tetrahydromethanopterin (methenyl-H_4_MPT^+^) is bound by the protein, bringing together FeGP and methenyl-H_4_MPT^+^. This is the active site that heterolytically cleaves molecular hydrogen into a proton and a hydride, whereby the hydride is stereospecifically transferred to the H*pro*-R position of the methylene carbon of methylene-H_4_MPT^238^ (Figure 13b). So far, computational models have predicted several iron–hydrogen species in the catalytic cycle, none of which could be experimentally verified. The use of pH_2_ in hyperpolarization experiments has recently changed this.^151^

**Figure 14:**
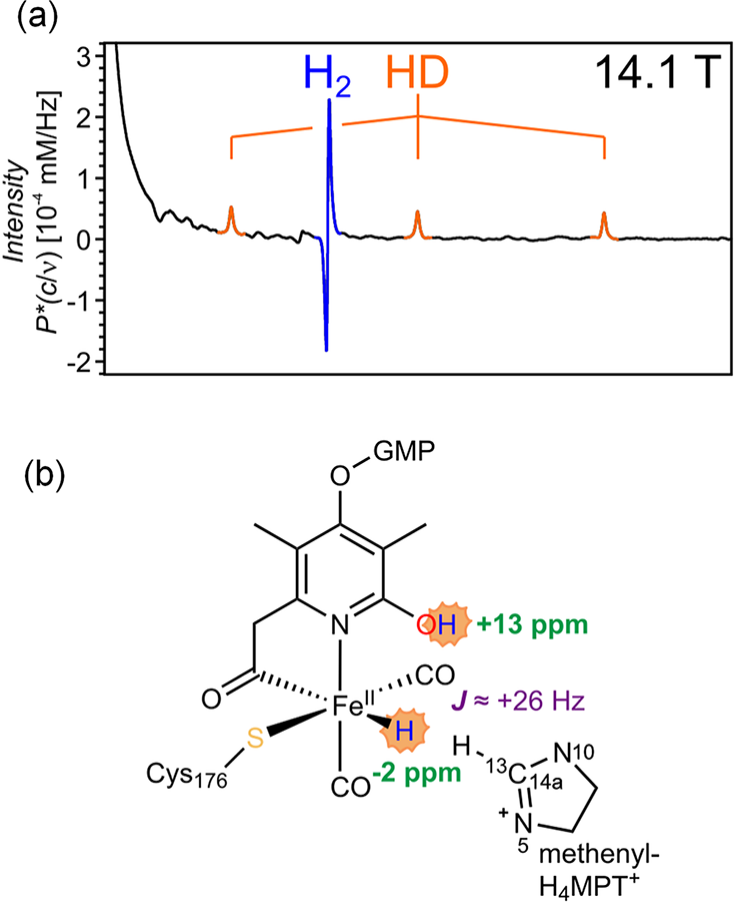
Using PHIP to reveal hitherto unobservable intermediates of [Fe] hydrogenase. (a) Hyperpolarized signals were observed when pH_2_ was supplied to an enzymatic solution of the hydrogenase containing methenyl-H4MPT+. A PNL effect and oneH-PHIP hyperpolarized HD signals demonstrate the activation of pH_2_ by hydrogenase. (b) The reaction intermediate was determined using observed NMR parameters and analysis of chemical exchange, and it was derived from a crystal structure quantum mechanical optimization of the structure. *Adapted from Kaltschnee et al*.^151^*Copyright 2024 Springer Nature. This publication is licensed under CC-BY 4.0*.

When pH_2_ was supplied to an aqueous buffer containing [Fe]-hydrogenase and methenyl-H_4_MPT^+^, the appearance of a hyperpolarized PNL (section 2.2) as well as HD and HDO NEPTUN PHIP signals (section 2.4) was observed (Figure 14a).^151^ The former state is created when pH_2_ reversibly binds to the enzyme. In addition, hyperpolarized HD signals, first observed with iridium catalysts in the context of the NEPTUN effect,^239^ were also observed in the presence of hydrogenase when the buffer was partially deuterated. This finding further indicates an isotope exchange with the solvent. In addition, rapid exchange between an enzyme-bound ensemble of hydrogen where both hydrogens are distinguishable, and a state where both hydrogens are indistinguishable on the NMR time scale, needs to occur. An estimate for the lifetime range of 1–100 μs was found, and chemical shifts and ^1^H–^1^H *J*-coupling constants between these hydrogens were estimated. Optimized structural models based on the X-ray crystal structure of the hydrogenase allowed for the computation of ^1^H chemical shifts and ^1^H–^1^H *J*-coupling constants for the hydrogen atoms within the active site and correlation with the experimental observations. Considering all this, an intermediate could be identified that had only been predicted previously (Figure 14b).^240^ The optimized structure reveals the presence of an iron hydride and the involvement of the oxopyridine site during the activation process. The determined intermediate supports the previously theoretically predicted process during which an oxo-pyridine moiety serves as a base for hydrogen activation during the catalytic process.

With this demonstration, the use of pH_2_-enhanced magnetic resonance has evolved into a tool to study the biochemistry and catalysis of hydrogenases and to investigate so far undetectable reaction intermediates of this important class of enzymes. It is envisioned that the concept can be used to study additional hydrogen-activating enzymes, such as the other two types of hydrogenases and potentially nitrogenases.

## 3 Outlook

Almost 40 years after its first discovery, PHIP remains a source for ever-surprising novel applications and unique revelations, some of which are discussed in this review. In the wake of biomedical hyperpolarized MRI, recent discoveries of efficient ways to hyperpolarize ^13^C-labeled pyruvate led to its *in vivo* imaging demonstrations.^85,86,88,89^ The high levels of polarization achieved using PHIP led to observations of effects such as radio amplification by stimulated emission of radiation (RASER) and spin diffusion in the solid state that attracted attention beyond the chemical community.^58,241−247^ Continued exploration and development of PHIP has and will likely continue to yield tools and techniques that complement more traditional methods, which have their shortcomings.

One promising direction lies in the continued development of catalysts and reaction conditions that enable PHIP with catalytic systems previously considered incompatible with PHIP. The ability to perform PHIP in these settings can lead to more sustainable and versatile applications. Hence, the field of applied catalysis will be enriched among others through synergetic development with PHIP as it was when the homogeneous catalysts were tuned to reach exclusive *trans* hydrogenation for a direct hyperpolarization of fumarate,^44^ or when PHIP was demonstrated using homogeneous,^17−19^ heterogeneous,^15,42,43,248−268^ and metal-free^148,149,180,^ catalytic systems. Contrast agents produced by heterogeneous PHIP are currently progressing toward *in vivo* lung imaging.^47,48^

It is worth highlighting the innovative combination of hyperpolarization for boosting NMR sensitivity while maintaining its quantitative nature. Tessari and others proposed two compelling approaches to utilize SABRE in analytical chemistry. In one method, analytes are introduced and hyperpolarized via SABRE, enabling concentration evaluations with near-nanomolar sensitivity.^250−254^ This demonstrates the potential of SABRE as a powerful quantitative tool. The second approach focuses on hyperpolarizing the hydride signals of the catalyst, exploiting the chemical shifts induced by the coordination of trace analytes to the Ir-complex.^104,12,255−269^ This method transforms the hyperpolarized hydrides into dynamic probes for chemical analysis, opening avenues for studying even minute concentrations of analytes with precision.

Furthermore, integrating PHIP with advanced NMR techniques, such as ultrafast sequences and ultralow and high-field instrumentation, may allow for detecting and studying transient intermediates and reaction dynamics with unprecedented sensitivity, and spectral and temporal resolution.^259−263^ This can significantly enhance our understanding of complex chemical processes, lead to the discovery of new reaction pathways, and uncover details of catalytic mechanisms.

All discussed here was made possible by utilizing the correlation of spins in pH_2_ and its effects on the nuclear spin polarization of other interacting neighboring atomic nuclei. Observing nonequilibrium polarization with NMR provides chemical resolution, enabling chemical analysis on an atomic level. NMR, however, is relatively slow, although, in some cases, like in photo-PHIP, the time resolution was boosted while preserving the chemical resolution. For example, a similar boost in time resolution was achieved when photoinduced radical pairs could be generated with a short UV irradiation, resulting in time-resolved chemically induced dynamic nuclear polarization.^264^ Many methods discussed here are still under development and require significant expertise that currently limits their wider use. However, it is worth being aware of such effects, as, for example, they could provide invaluable information regarding the mobility of H_2_ on catalytic centers that are inaccessible to other methods, as was exemplified here with hydrogenase studies.
